# Synthesis and Redox Activity of Polyenaminones for Sustainable Energy Storage Applications

**DOI:** 10.3390/polym16192700

**Published:** 2024-09-24

**Authors:** Tomaž Kotnik, Svit Menart, Žan Adam, Jan Bitenc, Luka Ciber, Uroš Grošelj, Nejc Petek, Bogdan Štefane, Jurij Svete, Boštjan Genorio

**Affiliations:** 1 University of Ljubljana, Faculty of Chemistry and Chemical Technology, Večna pot 113, 1000 Ljubljana, Slovenia; tomaz.kotnik@ki.si (T.K.); svit.menart@ki.si (S.M.); za7814@student.uni-lj.si (Ž.A.); luka.ciber@fkkt.uni-lj.si (L.C.); uros.groselj@fkkt.uni-lj.si (U.G.); nejc.petek@fkkt.uni-lj.si (N.P.); bogdan.stefane@fkkt.uni-lj.si (B.Š.); 2 National Institute of Chemistry, Hajdrihova 19, 1000 Ljubljana, Slovenia; jan.bitenc@ki.si

**Keywords:** polyenaminones, β-keto enamine-linked conjugated polymers, transaminative polymerization, phenylenediamines, optical activity, redox activity

## Abstract

In the search for novel polymeric molecules that could be used as electroactive materials, seven novel polyenaminones were prepared in high yields by the transaminative polymerization of resorcinol-derived bis-enaminones with *m*- and *p*-phenylenediamine and with 2,5-diaminohydroquinone. The obtained polymers show very low solubility in organic solvents and absorb UV light and visible light at wavelengths below 500 nm. All the obtained polymeric products were tested for redox activity in a Li battery setup. The 2,5-diaminohydroquinone-derived compound showed the best redox activity, with a maximum capacity of 86 mAh/g and relatively good capacity retention, thus confirming the hydroquinone group as the primary redox-active group. Other potential redox-active groups, such as resorcinol and conjugated carbonyls, showed limited activity, while variations in the phenylene groups and the substitution of phenolic groups in the resorcinol residue did not impact the electrochemical activity of the polymers. Their electrochemical properties, together with their previously established chemical recyclability, make polyenaminones promising scaffolds for the development of materials for sustainable energy storage applications.

## 1. Introduction

The development of synthetic polymers began relatively late on the chemistry timeline, with the first reported examples at the end of 19th century [1,2]. Considering this, it is fascinating that in such a brief time, modern life without synthetic polymers became unimaginable. As human civilization progresses rapidly, novel materials with specific properties are constantly needed to meet the requirements of advanced technologies [1,2]. Because of the growing demands for novel materials and to prevent future ecological crises, great efforts are put into development of novel synthesis routes for degradable polymers with desired properties [3,4] that are compliant with the requirements of sustainable development [5,6].

Carbon-based energy commodities (coal, natural gas, and petroleum) are the basis for producing most of the world’s energy today. Although various policies advocate energy from renewable sources, the use of renewable energy is still quite limited today [7,8]. In this regard, the scientific community is developing renewable-energy-based technologies and technologies that reduce the impact of greenhouse gases on climate change. Energy storage systems and, in particular, Li-ion batteries, are being accepted worldwide as alternatives to reduce the carbon footprint. However, the drawbacks of redox-active inorganic materials used in batteries are mainly related to the use of critical raw materials such as cobalt, lithium, nickel, graphite, etc. Numerous studies suggest that inorganic redox-active materials could be replaced by renewable organic materials. Today, organic semiconductors, and especially redox-active materials, are the basis for the transition to sustainable renewable energy sources [9,10,11]. Among different redox-active organic molecules, quinone (and related structural elements) or its reduced form, hydroquinone, offer interesting possibilities due to their high capacity and redox potential [12,13,14,15].

Enaminones are vinylogous amides in which a C=C double bond is inserted into a C–N bond of the amide [16], which are used as versatile difunctional reagents in organic synthesis [17,18,19,20]. Recently, there has been a notable increase in research interest in the enaminone motif in the field of materials science, particularly for the synthesis of covalent organic frameworks (COF)s [21]. Enaminone-linked COFs were employed for photocatalytic applications, methane storage, and chiral induction. However, the polymeric analogs, i.e., polyenaminones, also known as β-keto enamine-linked conjugated polymers, have been little studied [22]. Polyenaminones are generally available by polycondensation between diamines and various dielectrophiles, such as 1,3-dicarbonyls and analogues [23,24,25,26,27,28,29], diynones [30,31,32], and bis(*N*,*N*-disubstituted) enaminones [33,34,35,36]. Being conjugated enaminone push–pull systems, polyenaminones absorb UV and visible light. They also exhibit redox activity [36] and film-forming properties [34], and they are degradable and recyclable under mild conditions [29,35].

**Figure 1:**
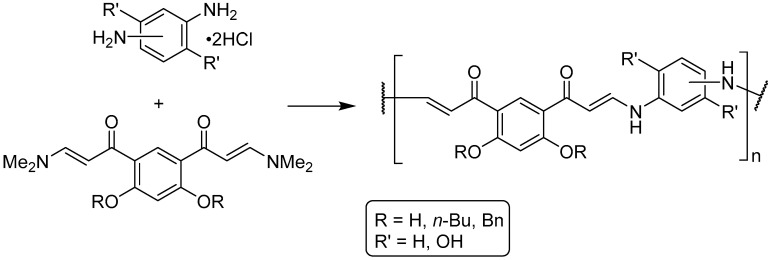
Preparation of polyenaminones from resorcinol (**1**)-derived bis-enaminones and phenylenediamines.

In the continuation of our research on polyenaminones [34,35,36], we recently focused our attention on redox-active polyenaminones that could be useful in energy storage and conversion systems. After initial encouraging results on cyclohexanedione-based polyenaminones [36], the studies were extended to resorcinol- and hydroquinone-based polyenaminones that could potentially improve the stability and increase the capacity of cathode materials for Li-ion and “beyond lithium” battery technologies (Na, Mg, Al, Ca batteries). Herein, we report on the synthesis, optical properties, and redox activity of a new family of polyenaminones, which are easily accessible from resorcinol and phenylenediamines (Figure 1).

## 2. Materials and Characterizations

### 2.1. Materials

All solvents and reagents were used as received. Resorcinol (**1**, ≥99.0%), *m*-phenylenediamine dihydrochloride (**4a**) (≥99.0%), *p*-phenylenediamine dihydrochloride (**4b**) (≥99.0%), 2,5-diaminohydroquinone dihydrochloride (**4c**) acetic anhydride (≥98.0%), zinc chloride (≥99.0%), potassium carbonate (99%), methyl iodide (99.5%), benzyl bromide (98%), 1-butyl bromide (99%), and *N*,*N*-dimethylformamide dimethylacetal (DMFDMA, for synthesis, ≥96%) are commercially available from Sigma-Aldrich (St. Louis, MO, USA).

Melting points (mps) were determined on a Kofler micro hot stage and on a Mettler Toledo MP30 automated melting point system (Mettler Toledo, Columbus, OH, USA). The NMR spectra were recorded in DMSO-*d*_6_ as deuterated solvent using Me_4_Si as the internal standard on Bruker Avance DPX 300, Bruker Avance III UltraShield 500, and Bruker Avance Neo 600 instruments (Bruker, Billerica, MA, USA) at 300, 500, and 600 MHz for ^1^H and at 75.5, 126, and 151 MHz for ^13^C nucleus, respectively. Chemical shifts (δ) are given in ppm relative to Me_4_Si as internal standard (δ = 0 ppm) and vicinal coupling constants (*J*) are given in hertz (Hz). UV-vis spectra were recorded in MeOH using a Varian Cary Bio50 UV-Visible Spectrophotometer (Agilent Technologies, Santa Clara, CA, USA). Fourier-transform infrared (FT-IR) spectra were obtained on a Bruker FTIR Alpha Platinum spectrophotometer (Bruker, Billerica, MA, USA) using attenuated total reflection (ATR) sampling technique. HRMS spectra were recorded on an Agilent 6224 time-of-flight (TOF) mass spectrometer equipped with a double orthogonal electrospray source under atmospheric pressure ionization (ESI) coupled to an Agilent 1260 high-performance liquid chromatograph (HPLC) (Agilent Technologies, Santa Clara, CA, USA). Microanalyses for C, H, and N were obtained on a Perkin-Elmer CHNS/O Analyzer 2400 Series II (PerkinElmer, Waltham, MA, USA).

Scanning electron microscopy (SEM) was used to investigate the morphological and topological features of the materials. Micrographs were taken on a Zeiss ULTRA plus SEM (Zeiss, Jena, Germany) instrument. Samples were adhered to the conductive carbon tape placed on the aluminum SEM holder and Au/Pd (80/20) coated, with a nominal thickness of 20 nm, using a Qurum Q150T ES turbomolecular pumped coater (AGC, Charleroi, Belgium). SEM images were taken at 2 kV using SE2 detector at WD 5.5 mm. SEM images were analyzed for particle size distribution with AxioVision 4.8 software (Informer Technologies, Inc., Los Angeles, CA, USA). Nitrogen physisorption isotherms were obtained on an ASAP 2020 Micromeritics instrument. Specific surface areas (SSA)s were determined using the Brunauer–Emmett–Teller theory. Samples were degassed under vacuum at 30 °C for 1 h. Organic cathodes were prepared by mixing polymers with Printex XE2 carbon black and PTFE binder (60 wt.% water dispersion) in a 60:30:10 weight ratio. All of the components and isopropanol (Sigma-Aldrich, St. Louis, MO, USA) were added into a stainless steel jar and homogenized for 30 min on a Retsch PM100 (Retsch, Haan, Germany) ball mill at 300 rpm. Composite matrices were then rolled in between a glass plate and a sheet of baking paper to give self-standing electrodes. Subsequently, 12 mm self-standing electrodes were cut, dried, and transferred into the Ar-filled glovebox. The average loading on the electrode was around 2.5 mg of active material per cm^2^. Battery cells were assembled in an argon-filled glovebox with water and oxygen levels below 1 ppm. Swagelok-type battery cells were assembled using the aforementioned electrodes, a 13 mm glass fiber separator (Whatman GF/A, Whatman plc, Maidstone, Kent, UK), and freshly rolled lithium (12 mm diameter, Sigma-Aldrich, St. Louis, MO, USA). The electrolyte used was a 1 M solution of bis(trifluoromethane)sulfonimide lithium salt in a mixture of dry 1,3-dioxolane and dimethoxyethane (1 M LiTFSI in DOL + DME, Sigma-Aldrich, St. Louis, MO, USA). Electrochemical measurements were performed at room temperature (25 °C) using a potentiostat/galvanostat VMP3 (Bio-Logic, Seyssinet-Pariset, France). The batteries were galvanostatically cycled between 1.5 and 3.5 V vs. Li/Li+ at a current density of 50 mA/g based on the mass of the tested polymer.

### 2.2. Synthesis of 4,6-Diacetylresorcinol (***2a***) [37,38]

This compound was prepared according to a slightly modified procedure from the literature [37]. A mixture of resorcinol (**1**) (10 g, 91 mmol), acetanhydride (37 mL, 182 mmol), and ZnCl_2_ (10 g, 73 mmol) was placed in a 250 mL flask, equipped with a reflux condenser. The mixture was heated at 140 °C for 30 min, and then cooled to room temperature. Water (20 mL) was added slowly (exothermic reaction) through a reflux condenser and the reaction mixture was heated to boiling point. Next, ethanol was added until complete dissolution of the precipitate (approx. 4 mL). The reaction mixture was cooled to room temperature and the precipitate was collected by filtration to give **2a**. Yield: 30.5 g (88%) of white solid; mp = 179–181 °C, mp = 178–179 °C [37], mp = 182 °C [38]. FT-IR (ATR): ν_max_ 3407, 3218, 3085, 1621 (C=O), 1488, 1336, 1254, 1046, 951, 833, 660 cm^−1^. ^1^H NMR (500 MHz; CDCl_3_): *δ* 2.63 (6H, s), 6.43 (1H, s), 8.21 (1H, s), 12.93 (2H, s). ^13^C NMR (126 MHz; CDCl_3_): *δ* 202.5, 169.0, 136.3, 113.7, 105.0, 26.1. *m/z* (HRMS): 195.0651 (MH^+^). C_10_H_11_O_4_ requires *m/z* 195.0652. Spectral data are in agreement with the literature data [37].

### 2.3. General Procedure for the Synthesis of Dialkylated 4,6-Diacetylresorcinols ***2b*** and ***2c*** [39]

Compounds **2b** and **2c** were prepared according to a modified procedure from the literature [39]. While cooling in an ice-bath, alkyl halide (20 mmol) was slowly (portion-wise) added to a mixture of diketone **2a** (1.94 g, 10 mmol), anh. DMF (20 mL), and K_2_CO_3_ (2.76 g, 20 mmol) and the mixture was stirred at 20–80 °C for 5–24 h. The precipitated product was collected by filtration to give **2b** and **2c**. The compounds described below were prepared in this manner.

#### 2.3.1. 1,1′-(4,6-Dibutoxy-1,3-phenylene)bis(ethan-1-one) (**2b**)

Prepared from **2a** (780 mg, 4 mmol), butyl bromide (1.2 mL, 1.5 g, 11 mmol), and K_2_CO_3_ (3 g, 22 mmol), 80 °C, 24 h. Yield: 725 mg (72%) of brownish solid; mp = 85–88 °C. FT-IR (ATR): ν_max_ 2956, 2870, 1663 (C=O), 1581, 1459, 1355, 1277, 1224, 1192, 1061, 1020, 956, 817 cm^−1^. ^1^H NMR (500 MHz; CDCl_3_): *δ* 1.00 (6H, t, *J* = 7.4 Hz), 1.54 (4H, br quintet, *J* = 7.4 Hz), 1.87 (4H, br quintet, *J* = 7.0 Hz), 2.57 (6H, s), 4.10 (4H, t, *J* = 6.4 Hz), 6.39 (1H, s), 8.33 (1H, s). ^13^C NMR (126 MHz; CDCl_3_): *δ* 13.9, 19.5, 31.2, 31.9, 68.9, 95.9, 120.9, 134.9, 163.3, 197.3. *m/z* (HRMS): 307.1902 (MH^+^). C_18_H_27_O_4_ requires *m/z* 307.1904.

#### 2.3.2. 1,1′-(4,6-Dibenzyloxy-1,3-phenylene)bis(ethan-1-one) (**2c**) [39]

Prepared from **2a** (2.0 g, 10.4 mmol), benzyl bromide (2.4 mL, 3.55 g, 21 mmol), and K_2_CO_3_ (2.875 g, 21 mmol), 20 °C, 5 h. Yield: 3.12 mg (81%) of light brownish solid; mp = 124–126 °C, mp = 126–127 °C [39]. FT-IR (ATR): ν_max_ 3061, 3003, 2996, 1656 (C=O), 1579, 1558, 1500, 1427, 1387, 1353, 1274, 1223,1188, 1055, 1005, 961, 822, 735, 695,605 cm^−1^. ^1^H NMR (500 MHz; CDCl_3_): *δ* 2.54 (6H, s), 5.15 (4H, s), 6.54 (1H, s), 7.37–7.41 (10H, m), 8.34 (1H, s). ^13^C NMR (126 MHz; CDCl_3_): *δ* 31.9, 71.2, 97.8, 121.6, 127.6, 128.7, 129.0, 134.9, 135.5, 162.5, 197.2. *m/z* (HRMS): 375.1586 (MH^+^). C_24_H_23_O_4_ requires *m/z* 375.1591.

### 2.4. General Procedure for the Synthesis of Bis-Enaminones ***3a**–**c***

Enaminones **3a**–**c** were prepared following slightly modified procedure from the literature for the synthesis of compound **3a** [40]. A mixture of diketone **2** (10 mmol), anh. xylene (30 mL), and DMFDMA (3.5 mL, 25 mmol) was heated under reflux for 24 h. The reaction mixture was cooled to 0 °C (ice bath) and the precipitate was collected by filtration to give **3**. The compounds described below were prepared in this manner.

#### 2.4.1. (2*E*,2′*E*)-1,1′-(4,6-dihydroxy-1,3-phenylene)bis [3-(dimethylamino)prop-2-en-1-one] (**3a**) [40]

Prepared from **2a** (4.1 g, 21 mmol) and DMFDMA (6.1 mL, 44 mmol). Yield: 5.20 g (82%) of brown solid; mp = 196–199 °C, mp = 198–200 °C [40]. FT-IR (ATR): ν_max_ 2902, 2805, 1615 (C=O), 1528, 1432, 1366, 1279, 1187, 1104, 891, 785, 712, 602 cm^−1^. ^1^H NMR (600 MHz; CDCl_3_): *δ* 2.95 and 3.16 (12H, 2 br s, 1:1), 5.63 (2H, d, *J* = 12.1 Hz), 6.35 (1H, s), 7.82 (2H, d, *J* = 12.1 Hz), 8.04 (1H, s), 14.73 (2H, s). ^13^C NMR (151 MHz, CDCl_3_) δ 190.5, 168.6, 154.5, 130.4, 113.1, 104.6, 89.3, 45.5, 37.4. *m/z* (HRMS): 305.1493 (MH^+^). C_16_H_21_N_2_O_4_ requires *m/z* 305.1496. Spectral data are in agreement with the literature data [40].

#### 2.4.2. (2*E*,2′*E*)-1,1′-(4,6-dibutoxy-1,3-phenylene)bis [3-(dimethylamino)prop-2-en-1-one] (**3b**)

Prepared from **2b** (602 mg, 2.4 mmol) and DMFDMA (0.8 mL, 6 mmol). Yield: 800 mg (80%) of beige solid; mp = 145–147 °C. FT-IR (ATR): ν_max_ 2947, 2868, 1637 (C=O), 1589, 1534, 1414, 1342, 1257, 1226, 1163, 1096, 1058, 1023, 968, 810, 782, 748, 666, 609 cm^−1^. ^1^H NMR (300 MHz; CDCl_3_): *δ* 0.96 (6H, t, *J* = 7.4 Hz), 1.51 (4H, br sextet, *J* = 7.3 Hz), 1.79 (4H, br quintet, *J* = 7.0 Hz), 2.95 (12H, br s), 4.03 (4H, t, *J* = 6.4 Hz), 5.67 (2H, d, *J* = 12.7 Hz), 6.42 (1H, s), 7.56 (2H, d, *J* = 12.7 Hz), 7.90 (1H, s). ^13^C NMR (75.5 MHz; DMSO-d_6_): *δ* 13.9, 19.4, 31.3, 45.0, 68.6, 97.4, 98.2, 123.4, 126.0, 132.3, 153.9, 159.6 (found: C, 68.81; H, 8.62; N, 6.27. C_24_H_36_N_2_O_4_·¼H_2_O requires C, 68.46; H, 8.74; N, 6.65%). *m/z* (HRMS): 417.2744 (MH^+^). C_24_H_37_N_2_O_4_ requires *m/z* 417.2748.

#### 2.4.3. (2*E*,2′*E*)-1,1′-(4,6-dibenzyloxy-1,3-phenylene)bis [3-(dimethylamino)prop-2-en-1-one] (**3c**)

Prepared from **2c** (1.88 g, 5 mmol) and DMFDMA (1.75 mL, 11.7 mmol). Yield: 2.06 g (84%) of beige solid; mp = 171–174 °C. FT-IR (ATR): ν_max_ 3484, 2938, 2877, 1635 (C=O), 1587, 1532, 1418, 1349, 1251, 1169, 1098, 989, 909, 845, 793, 730, 698, 658, 625 cm^−1^. ^1^H NMR (300 MHz; CDCl_3_): *δ* 2.72 and 2.98 (12H, 2s, 1:1), 5.09 (4H, s), 5.65 (2H, d, *J* = 12.7 Hz), 7.28–7.45 (10H, m), 7.54 (2H, d, *J* = 12.4 Hz), 7.95 (1H, s). ^13^C NMR (126 MHz; CDCl_3_): *δ* 35.0, 37.2, 45.0, 71.0, 98.2, 99.4, 124.3, 127.6, 128.1, 128.7, 132.4, 154.4, 159.0. *m/z* (HRMS): 485.2430 (MH^+^). C_30_H_33_N_2_O_4_ requires *m/z* 485.2435.

### 2.5. Synthesis of Compounds ***5aa**–**5ac***, ***5ba***, ***5bb***, ***5ca***, and ***5cb***

General Procedure. A mixture of bis-enaminone monomer **3** (1 mmol), diamine dihydrochloride **4** (1 mmol), and methanol (10 mL) was shaken on an orbital shaker at room temperature for 24 h. The precipitate was collected by filtration, washed with methanol (2 mL), and air-dried to give **5**. The compounds described below were prepared in this manner.

#### 2.5.1. Poly{(*EZ*)-3-{[3-(λ^2^-azaneyl)phenyl]amino}-1-[2,4-dihydroxy-5-(3λ^3^-prop-2-enoyl)phenyl]prop-2-en-1-one} (**5aa**)

Prepared from **3a** (305 mg, 1 mmol) and 1,3-phenylenediamine dihydrochloride (**4a**) (182 mg, 1 mmol). Yield: 306 mg (95%) of dark ochre solid. FT-IR (ATR): ν_max_ 3067, 1628 (C=O), 1593, 1538, 1481, 1267, 1183, 1161, 993, 838, 713 cm^−1^. ^1^H NMR (500 MHz; DMSO-d_6_): *δ* 6.02–8.65 (10H, m), 10.39–10.74 and 11.38–11.63 (2H, 2m, 2:1), 13.67–15.71 (2H, m). ^13^C NMR (126 MHz; DMSO-d_6_): *δ* 190.4, 190.1, 189.8, 175.6, 166.4, 159.4, 157.8, 156.8, 147.1, 146.5, 141.9, 141.4, 131.3, 130.3, 126.6, 124.0, 119.2, 116.6, 116.6, 113.2, 112.2, 112.0, 112.0, 104.6, 39.5 (multiple peaks for each type of carbon nuclei are due to slow isomerization around the C=C double bonds). Found: C, 62.59; H, 4.39; N, 7.70. C_18_H_14_N_2_O_4_·⅔HCl requires C, 62.37; H, 4.27; N, 8.08%. λ_max_ (MeOH)/nm 420.

#### 2.5.2. Poly{(*EZ*)-3-{[4-(λ^2^-azaneyl)phenyl]amino}-1-[2,4-dihydroxy-5-(3λ^3^-prop-2-enoyl)phenyl]prop-2-en-1-one} (**5ab**)

Prepared from **3a** (305 mg, 1 mmol) and 1,4-phenylenediamine dihydrochloride (**4b**) (182 mg, 1 mmol). Yield: 322 mg (100%) of red-brown solid. FT-IR (ATR): ν_max_ 3233, 1605 (C=O), 1536, 1485, 1267, 1178, 817, 714 cm^−1^. ^1^H NMR (300 MHz; DMSO-d_6_): *δ* 5.98–8.52 (10H, m), 10.21–10.80 and 11.53–11.73 (2H, 2m, 3:2), 13.83–15.83 (2H, m). ^13^C NMR (126 MHz; DMSO-d_6_): *δ* 39.5, 104.6, 106.7, 112.0, 117.6, 119.2, 125.4, 126.6, 126.9, 133.0, 133.4, 140.8, 145.6, 159.3, 166.4, 169.1, 175.6 (multiple peaks for each type of carbon nuclei are due to slow isomerization around the C=C double bonds). Found: C, 63.20; H, 4.34; N, 7.38. C_18_H_14_N_2_O_4_·½HCl requires C, 63.49; H, 4.29; N, 8.24%. λ_max_ (MeOH)/nm 455.

#### 2.5.3. Poly{(*EZ*)-3-{[4-(λ^2^-azaneyl)-2,5-dihydroxyphenyl]amino}-1-[2,4-dihydroxy-5-(3λ^3^-prop-2-enoyl)phenyl]prop-2-en-1-one} (**5ac**)

Prepared from **3a** (76 mg, 0.25 mmol) and 2,5-diaminohydroquinone dihydrochloride (**4c**) (53 mg, 0.25 mmol). Yield: 96 mg (100%) of red-brown solid. FT-IR (ATR): ν_max_ 3344, 3153, 1614 (C=O), 1526, 1493, 1364, 1269, 1177, 1105, 839, 784, 711 cm^−1^. ^1^H NMR (500 MHz; DMSO-d_6_): *δ* 6.00–6.47 (2H, m), 6.80–7.25 (3H, m), 7.54–8.51 (5H, m), 9.62–10.20 (2H, m), 11.56–11.90 (1H, m), 13.16–14.38 (1H, m), 14.98–15.80 (1H, m). ^13^C NMR (151 MHz; DMSO-d_6_): *δ* 189.2, 178.4, 175.9, 168.0, 159.2, 157.8, 156.7, 155.4, 153.6, 153.3, 151.3, 130.5, 126.6, 124.0, 112.3, 112.2, 112.0, 107.5, 104.7, 103.5, 101.1, 94.6, 92.9, 89.0 (multiple peaks for each type of carbon nuclei are due to slow isomerization around the C=C double bonds). Found: C, 57.41; H, 3.80; N, 7.13. C_18_H_14_N_2_O_6_·⅔HCl requires C, 57.10; H, 3.90; N, 7.40%. λ_max_ (MeOH)/nm 340, 380.

#### 2.5.4. Poly{(*EZ*)-3-{[3-(λ^2^-azaneyl)phenyl]amino}-1-[2,4-bis(butoxy)-5-(3λ^3^-prop-2-enoyl)phenyl]prop-2-en-1-one} (**5ba**)

Prepared from **3b** (208 mg, 0.5 mmol) and 1,3-phenylenediamine dihydrochloride (**4a**) (91 mg, 0.5 mmol). Yield: 189 mg (88%) of ochre solid. FT-IR (ATR): ν_max_ 2954, 2929, 2869, 1630 (C=O), 1576, 1458, 1253, 1177, 1156, 991, 785, 734, 681 cm^−1^. ^1^H NMR (500 MHz; DMSO-d_6_): *δ* 0.77–1.06 (6H, m), 1.17–1.37 (2H, m), 1.40–1.60 (3H, m), 1.68–1.90 (3H, m), 3.93–4.32 (4H, m), 6.09–6.47 (2H, m), 6.60–8.37 (10H, m), 9.81–9.97 and 11.83–12.01 (2H, 2m, 2:3). ^13^C NMR (126 MHz; DMSO-d_6_): *δ* 13.3, 13.7, 18.2, 18.8, 18.9, 30.6, 31.9, 68.4, 68.7, 77.2, 83.3, 95.8, 96.2, 97.8, 98.7, 106.7, 108.6, 108.9, 110.8, 119.5, 121.5, 130.9, 141.7, 141.9, 153.8, 157.0, 157.5, 157.9, 170.8, 178.3 (multiple peaks for each type of carbon nuclei are due to slow isomerization around the C=C double bonds). Found: C, 69.74; H, 7.13; N, 6.07. C_26_H_30_N_2_O_4_·⅔H_2_O requires C, 69.93; H, 7.07; N, 6.27%. λ_max_ (MeOH)/nm 395.

#### 2.5.5. Poly{(*EZ*)-3-{[4-(λ^2^-azaneyl)phenyl]amino}-1-[2,4-bis(butoxy)-5-(3λ^3^-prop-2-enoyl)phenyl]prop-2-en-1-one} (**5bb**)

Prepared from **3b** (208 mg, 0.5 mmol) and 1,4-phenylenediamine dihydrochloride **4b** (91 mg, 0.5 mmol). Yield: 199 mg (92%) of brown solid. FT-IR (ATR): ν_max_ 2954, 2929, 2889, 1622 (C=O), 1584, 1520, 1462, 1351, 1253, 1164, 963, 814, 784, 685 cm^−1^. ^1^H NMR (500 MHz; DMSO-d_6_): *δ* 0.77–1.06 (6H, m), 1.17–1.37 (2H, m), 1.40–1.60 (3H, m), 1.68–1.90 (3H, m), 3.93–4.32 (4H, m), 6.09–6.47 (2H, m), 6.60–8.37 (10H, m), 9.81–9.97 and 11.83–12.01 (2H, 2m, 2:3). ^13^C NMR could not be recorded due to extremely low solubility of compound **5bb** in DMSO-d_6_ (found: C, 70.03; H, 6.88; N, 6.22. C_26_H_30_N_2_O_4_·⅔H_2_O requires C, 69.93; H, 7.07; N, 6.27%). λ_max_ (MeOH)/nm 425.

#### 2.5.6. Poly{(*EZ*)-3-{[3-(λ^2^-azaneyl)phenyl]amino}-1-[2,4-bis(benzyloxy)-5-(3λ^3^-prop-2-enoyl)phenyl]prop-2-en-1-one} (**5ca**)

Prepared from **3c** (480 mg, 1 mmol) and 1,3-phenylenediamine dihydrochloride **4a** (182 mg, 1 mmol). Yield: 420 mg (84%) of brown solid. FT-IR (ATR): ν_max_ 3337, 3029, 1629 (C=O), 1579, 1494, 1454, 1255, 1156, 997, 729, 693 cm^−1^. ^1^H NMR (500 MHz; DMSO-d_6_): *δ* 5.15–5.43 (4H, m), 6.10–6.44 (2H, m), 6.68–7.59 (14H, m), 7.72–8.91 (4H, m), 9.91–10.05 and 11.84–11.93 (2H, 2m, 1:1). ^13^C NMR (126 MHz; DMSO-d_6_): *δ* 39.5, 70.1, 70.4, 70.8, 98.7, 99.0, 99.5, 127.1, 127.3, 127.7, 127.9, 128.0, 128.1, 128.1, 128.5, 128.6, 128.6, 128.7, 136.3, 141.6, 144.2, 144.5, 161.0, 161.2, 161.6, 188.4 (multiple peaks for each type of carbon nuclei are due to slow isomerization around the C=C double bond). Found: C, 74.28; H, 5.08; N, 5.43. C_32_H_26_N_2_O_4_·¾H_2_O requires C, 74.48; H, 5.37; N, 5.43%. λ_max_ (MeOH)/nm 395.

#### 2.5.7. Poly{(*EZ*)-3-{[4-(λ^2^-azaneyl)phenyl]amino}-1-[2,4-bis(benzyloxy)-5-(3λ^3^-prop-2-enoyl)phenyl]prop-2-en-1-one} (**5cb**)

Prepared from **3c** (480 mg, 1 mmol) and 1,4-phenylenediamine dihydrochloride **3b** (182 mg, 1 mmol). Yield: 455 mg (91%) of ochre solid. FT-IR (ATR): ν_max_ 3031, 2917, 2850, 1622 (C=O), 1585, 1522, 1468, 1349, 1257, 1201, 1161, 1109, 1003, 815, 878, 730, 695 cm^−1^. ^1^H NMR (500 MHz; DMSO-d_6_): *δ* 5.22–5.38 (4H, m), 5.66–5.73, 6.10–6.18, and 6.24–6.33 (2H, 3m, 19:47:34), 6.91–7.12 (2H, m); 7.21–8.21 (14H, m), 7.72–8.91 (4H, m), 9.84–10.01 and 11.97–12.05 (2H, 2m, 1:1). ^13^C NMR (126 MHz; DMSO-d_6_): *δ* 39.5, 70.1, 70.1, 70.4, 98.0, 98.2, 98.7, 99.1, 99.1, 117.4, 117.5, 117.6, 127.4, 127.7, 127.9, 128.0, 128.1, 128.5, 128.7, 135.9, 136.3, 136.4, 136.4, 136.5, 136.5, 136.7, 144.3, 144.4, 144.4, 158.9, 159.2, 159.3, 159.5, 159.9, 187.4, 187.9, 188.0, 188.2, 189.2 (multiple peaks for each type of carbon nuclei are due to slow isomerization around the C=C double bond). Found: C, 71.79; H, 5.69; N, 5.85. C_32_H_26_N_2_O_4_·¼Me_2_NH·¾H_2_O requires C, 72.13; H, 5.31; N, 5.82%. λ_max_ (MeOH)/nm 425.

## 3. Results

### 3.1. Synthesis

**Scheme 1:**
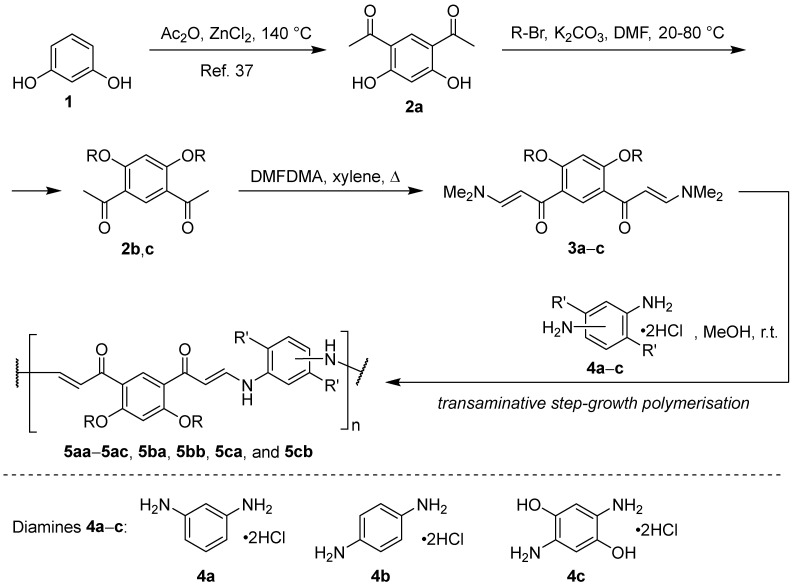
Synthesis of polymers **5aa**–**5ac**, **5ba**, **5bb**, **5ca**, and **5cb** by transamination of enaminones **3a**–**c** with diamine dihydrochlorides **4a**–**c** [37].

The starting diketones **2a**–**c** were prepared in 2–3 steps from resorcinol (**1**). The acetylation of **1** in the presence of anh. ZnCl_2_, following the procedure in the literature [37], gave 4,6-diacetylresorcinol (**2a**) in an 88% yield. The O-alkylation of **2a** with 1-butyl bromide and benzyl bromide in the presence of K_2_CO_3_ gave the diketones **2b** and **2c** in 72% and 81% yields, respectively (Scheme 1, Table 1, Entries 1–3). Heating diketones **2a**–**c** with 1.2 equiv. DMFDMA in xylene for 24 h then produced the respective bis-enaminone monomers **3a**–**c** in 62–84% yields (Scheme 1, Table 1, Entries 4–6). The bis-enaminones **3a**–**c** were then treated with equimolar amounts of phenylenediamines dihydrochlorides **4a**–**c** in methanol at room temperature for 24 h and the precipitated products **5aa**–**5ac**, **5ba**, **5bb**, **5ca**, and **5cb** were obtained by filtration in 84–100% yields (Scheme 1, Table 1, Entries 7–13). The reaction mechanism is explainable by step-growth polymerization through dimeric, trimeric, and oligomeric intermediates in the same manner as before for closely related transaminative polymerizations (Scheme 1) [20,34,35,36].

### 3.2. Characterization

The intermediates **2a**–**c** and **3a**–**c** and the title compounds **5aa**, **5ab**, **5ac**, **5ba**, **5bb**, **5ca**, and **5cb** were characterized by spectroscopic methods (^1^H NMR, ^13^C NMR, FTIR, HRMS, and UV-vis) and by elemental analysis for C, H, and N.

#### 3.2.1. Characterization by NMR

The ^1^H NMR and ^13^C NMR data for bis-enaminones **3a**–**c** and polyenaminones **5aa**–**5ac**, **5ba**, **5bb**, **5ca**, and **5cb** are in agreement with the data for closely related bis-enaminones and polyenaminones [34,35,36]. In the ^1^H NMR spectra of polyenaminones **5aa**, **5ab**, **5ba**, **5ca**, and **5cb**, the cumulative relative intensity of signals within the δ chemical shift frame 6–9 ppm corresponds to a total of ten aromatic and enamine CH=CH protons, while the cumulative relative intensity of signals within the δ chemical shift frame 10–12 ppm correspond to two NH protons. In the ^1^H NMR spectra of products **5aa** and **5ab**, the signals within the δ chemical shift frame 13.5–15 ppm correspond to the phenolic protons. Finally, the ^1^H NMR spectra of the O-alkylated compounds **3b**,**c**, **5ba**, **5ca**, and **5cb** exhibit the expected signals for the alkyl residues. Unfortunately, characterization of compound **5bb** by NMR was not possible due to its insolubility in DMSO-d_6_ and other organic solvents. For more details, see the Supporting Information.

#### 3.2.2. Characterization by FTIR

The FTIR spectra of compounds **2** (~1660 cm^−1^), **3** (~1635 cm^−1^), and **5** (~1615 cm^−1^) show absorption bands in a region between 1600 cm^−1^ and 1660 cm^−1^, which are characteristic for the C=O group of a conjugated ketone. The absorption bands around 2900 cm^−1^ in the spectra of compounds **2**, **3**, and **5** are in line with typical C–H and N–H absorption bands [32,33,34,35,36]. The broad absorption bands around 3000 cm^−1^ in the spectra of compounds **5** are in agreement with the N–H···O=C hydrogen bonding of compound **5** in the solid state.

#### 3.2.3. Characterization by SEM

**Figure 2:**
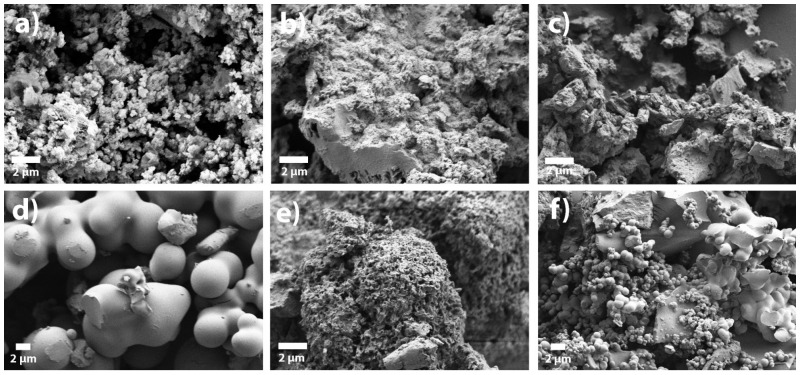
SEM images of the polymers (**a**) **5aa**, (**b**) **5ab**, (**c**) **5ac**, (**d**) **5ba**, (**e**) **5bb**, and (**f**) **5cb** were obtained using secondary electrons with an SE2 detector to examine their morphology.

The morphological properties (Figure 2) and particle size distribution (Figures S2–S7) of the title compounds **5aa**, **5ab**, **5ac**, **5ba**, **5bb**, and **5cb** were determined by scanning electron microscopy (SEM). Polymer **5aa** consists of agglomerated irregular particles approximately 100 nm in size (Figure S2). Polymer **5ab** exhibits a similar morphology, but with a wide particle size distribution, ranging from sub-100-nanometer particles to irregular chunks several micrometers in size (Figure S3). Polymer **5ac** also has a broad size distribution, predominantly featuring sharp-edged particles between 1 and 5 μm (Figure S4). Notably, polymer **5ba** consists of round-shaped merged structures approximately 10 μm and larger (Figure S5). Similarly shaped particles are observed in polymer **5cb**, but with smaller sizes and a wider distribution (Figure S7). Polymer **5bb** has the most porous structure, with agglomerates 5 μm and larger (Figure S6) consisting of interconnected small particles forming a highly porous network. For electrochemical applications, high-surface-area porous materials are desired to allow the electrolyte to access the polymer surfaces and utilize surface-redox-active functional groups. Polymers **5aa**, **5ab**, **5ac**, **5ba**, **5bb**, and **5cb** were tested for their specific surface area by N_2_ adsorption (BET experiment). The results revealed 68.3 m^2^/g, 3.8 m^2^/g, 0.1 m^2^/g, 1.4 m^2^/g, and 0.3 m^2^/g for **5aa**, **5ab**, **5ac**, **5bb**, and **5cb**. The specific surface area of **5ba** could not be measured due to its extremely low size. In this respect, materials **5aa**, **5ab**, and **5bb** exhibit the most promising morphologies in terms of specific surface area.

#### 3.2.4. Characterization by UV-Vis Spectroscopy

**Figure 3:**
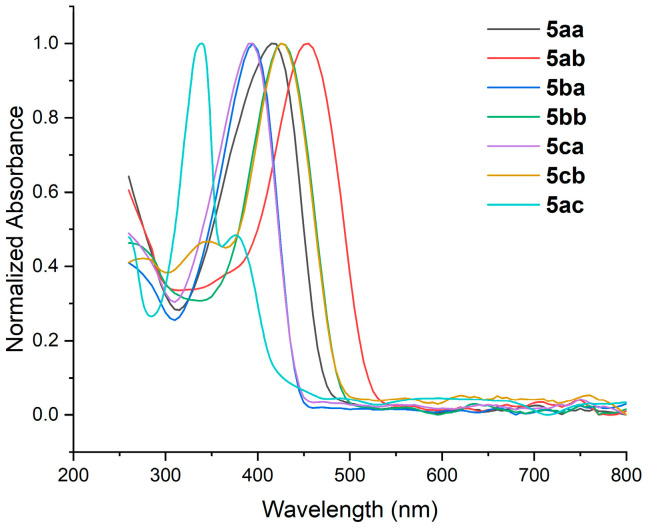
Normalized UV-vis spectra of compounds **5aa** (▬), **5ab** (▬), **5ba** (▬), **5bb** (▬), **5ca** (▬), **5cb** (▬), and **5ac** (▬).

The UV-vis spectra of compounds **5** were measured in MeOH at 250–800 nm (Figure S1). The normalized UV-vis spectra of compounds **5** are shown in Figure 3. Compounds **5** showed absorption maxima between 338 nm (**5ac** ▬) and 455 nm (**5ab** ▬). The strongest absorption maxima of the resorcinol-phenylenediamine-derived polymers **5aa**, **5ab**, **5ba**, **5bb**, **5ca**, and **5cb** were at the borderline of visible light between 395 nm (**5ba** ▬, **5ca** ▬) and 455 nm (**5ab** ▬). Compounds **5ab**, **5bb**, **5cb** had para-substituted diamine moieties absorbed at wavelengths that were 30–40 nm longer (455, 425, and 425 nm, respectively) than those in compounds **5aa**, **5ba**, **5ca**, which had meta-substituted diamine moieties (420, 395, and 395 nm, respectively) (Figure UV-vis). These absorption spectral data were consistent with the data for closely related polymers [34,35,36], thus indicating interesting UV-light-shielding properties that might find use in various optical applications.

#### 3.2.5. Determination of Redox Activity

Polyenaminones **5aa**, **5ab**, **5ac**, **5ba**, **5bb**, and **5cb** were electrochemically characterized in a Li electrolyte to probe their redox activity, which is also the starting point for the development of other monovalent (Na, Li, K) and multivalent (Mg, Ca, Al) batteries [9]. Specifically, we aimed to elucidate the potential redox-active centers of **5ac**, **5ab**, and **5bb**. Polymer **5ac** has three possible redox-active centers: hydroquinone, resorcinol, and conjugated carbonyl. In contrast, **5ab** contains only resorcinol and conjugated carbonyl, while **5bb** has only conjugated carbonyl. Despite these differences, the basic backbone structure of the polymers is quite similar.

**Figure 4:**
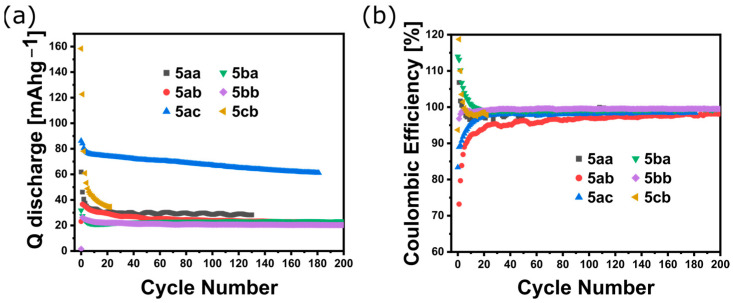
Specific capacity (**a**) and Coulombic efficiency (**b**) of the polymers **5aa**, **5ab**, **5ac**, **5ba**, **5bb**, and **5cb**.

The redox activity of all the materials was investigated in a Li-battery setup, with the polymers serving as cathode materials and Li metal as the anode. From the galvanostatic tests, the capacity versus the cycle number was extracted, as shown in Figure 4. Compound **5ac** exhibited an initial capacity of 86 mAh/g, which gradually faded to approximately 70 mAh/g after several cycles before stabilizing. In contrast to **5ac**, compound **5ab**, lacking a hydroquinone redox center, showed almost no redox activity, with a maximum capacity of 37 mAh/g. This suggests that the resorcinol group undergoes irreversible oxidation, rendering it ineffective for reversible cycling [41]. A similar trend was observed for **5aa**, which also contains a resorcinol center but has a 1,3-phenylene moiety instead of 1,4-phenylene, with an initial specific capacity of 62 mAh/g that rapidly drops to around 30 mAh/g. The final polymer in this sequence, **5bb**, has a resorcinol center protected by butyl groups and no hydroquinone redox center. It contains only a potentially redox-active conjugated carbonyl sequence. The electrochemical results showed a maximum capacity of 27 mAh/g, which indicates that the material has limited redox activity. Our previous testing of plain Printex carbon electrodes showed that the capacitance contribution of conductive carbon black inside the electrode should be around 15 mAh/g [42]. Similar electrochemical results were also observed for **5ba**, which delivered a maximum capacity of 32 mAh/g. Interestingly, **5cb** delivered the highest initial specific capacity of all the tested materials (158 mAh/g), which rapidly faded to 35 mAh/g. This behavior could be explained by initial redox-mediated cleavage of the benzyl ether groups, which afterwards show no redox activity [43]. Our results clearly indicate that the hydroquinone group in **5ac** is the primary redox-active group, while electrodes containing other redox-active groups such as resorcinol and conjugated carbonyls exhibit no significant redox activity, instead showing capacitive charge/discharge behavior of the carbon black additive and pseudocapacitance of the polymer. Furthermore, variations in the phenylene structure do not impact the electrochemical activity of the polymers. This conclusion is further supported by the Coulombic efficiency of compounds **5ac**, **5ab**, and **5bb**, which vary significantly and suggest possible side electrochemical reactions (Figure 4).

**Figure 5:**
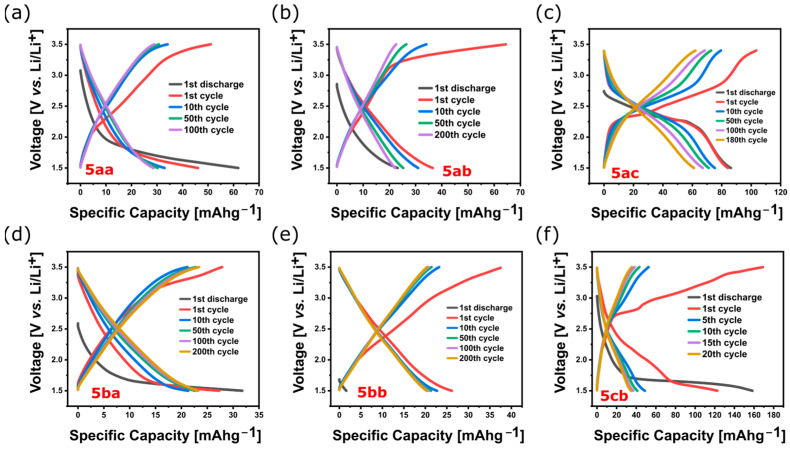
Galvanostatic charge/discharge curves of compounds of the polymers (**a**) **5aa**, (**b**) **5ab**, (**c**) **5ac**, (**d**) **5ba**, (**e**) **5bb**, and (**f**) **5cb**.

The galvanostatic curves shown in Figure 5 further support the rationalization above. All of the tested polymers, except **5ac** and **5cb**, show almost no redox activity, and most of the exhibited capacity is probably attributable to the capacitance of the carbon black conductive additive. The comparison between **5cb** and **5bb**, which differ in resorcinol-protecting groups (benzyl vs. butyl, respectively), showed additional plateaus of **5cb** in initial cycles, which rapidly transformed into a single sloping curve. As mentioned earlier, we attributed its additional redox process to the redox-mediated deprotection of the benzyl groups (Figure 5) [43].

On the other hand, **5ac** demonstrated high capacity (Figure 5c), although this was still lower than the theoretical value of 146 mAh/g, which is common for organic cathodes due to factors such as active particle morphology and electrolyte accessibility. Compound **5ac** displayed reversible redox activity, with a sloping discharge plateau at around 2.4 V vs. Li/Li+. It reached a maximum capacity of 86 mAhg^−1^, with an average discharge voltage of 2.30 V vs. Li/Li+ and a capacity retention of 71% after 180 cycles. The relatively high reversibility observed is most likely due to the presence of hydroquinone redox centers.

**Figure 6:**
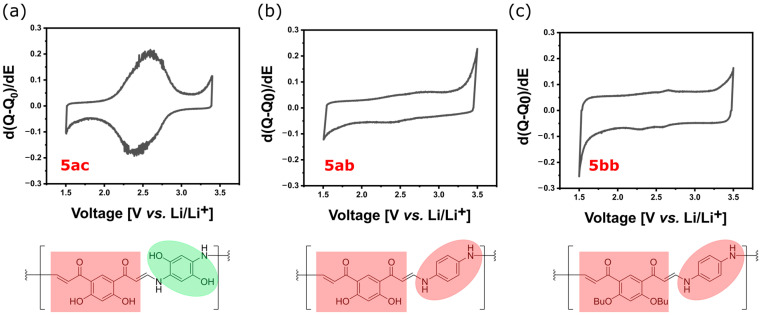
dQ/dE curves of compounds (**a**) **5ac**, (**b**) **5ab**, and (**c**) **5bb**.

This was further supported by the dQ/dE vs. potential (E) curves presented in Figure 6. In these curves, only **5ac** exhibited a redox peak, which appeared at a lower potential than the regular benzoquinone/hydroquinone (BQ/HQ) redox peak. This shift may be attributed to the donating effect of the -NH group on the phenylene ring [44]. The other two polymers, **5ab** and **5bb**, did not show any redox peaks in the dQ/dE curves.

Furthermore, when comparing the morphology (Figure 2) to the electrochemical results, the slightly less porous morphologies of **5ac** and **5ab** demonstrate good reversibility, although the specific capacities of both polymers are lower than their theoretical values (146 mAh/g). This is particularly noticeable for **5ac**, which showed redox activity through the hydroquinone center. Such low material utilization is common in Li–organic batteries and is typically due to restricted ionic and electronic transport within the organic materials [45]. This issue could be addressed through polymer engineering, in which the morphology can be optimized, for example, by the in situ addition of conductive nanofillers like CNTs [46].

## 4. Conclusions

Like other related polyenaminones, **5aa**, **5ab**, **5ac**, **5ba**, **5bb**, **5ca**, and **5cb** are also available through the acid-catalyzed transaminative polymerization between resorcinol-derived bis-enaminones **2a**–**c** and 1,3- and 1,4-phenylenediamines **3a** and **3b**. Monomer building blocks **2** and **3** are also accessible from biomass-derived cyclohexanediones, thus complying with the requirements of sustainable chemistry. Compounds **5** are optically active and exhibit strong absorption of UV-vis light below 500 nm, which makes them suitable for optical applications. Testing polyenaminones **5aa**, **5ab**, **5ac**, **5ba**, **5bb**, **5ca**, and **5cb** for redox activity revealed that the highest activity was by 2,5-diaminohydroquinone-derived compound **5ac**, with a maximum capacity of 86 mAh/g and relatively good capacity retention. This is in line with the premise that the hydroquinone moiety is the primary redox active group, while a significant drop in capacity after the first cycle suggests irreversible oxidation of the resorcinol moiety. The variation of other structural elements, such as the substitution of the phenolic hydroxy groups and the variation of the phenylenediamine moiety, did not affect the redox activity.

## Figures and Tables

**Table 1:** Experimental data for compounds **2a**–**c**, **3a**–**c**, **5aa**–**5ac**, **5ba**, **5bb**, **5ca**, and **5cb**.

Entry	Transformation	R	R′	Yield (%)
1	**1** → **2a**	-		88
2	**2a** → **2b**	*n*-Bu		72
3	**2a** → **2c**	CH_2_Ph		81
4	**2a** → **3a**	H		82
5	**2b** → **3b**	*n*-Bu		62
6	**2c** → **3c**	CH_2_Ph		84
7	**3a** + **4a** → **5aa**	H	H	95
8	**3a** + **4b** → **5ab**	H	H	100
9	**3a** + **4c** → **5ac**	H	OH	100
10	**3b** + **4a** → **5ba**	*n*-Bu	H	88
11	**3b** + **4b** → **5bb**	*n*-Bu	H	92
12	**3c** + **4a** → **5ca**	CH_2_Ph	H	84
13	**3c** + **4b** → **5cb**	CH_2_Ph	H	91

## Data Availability

The data presented in this study are available in the main manuscript and in the Supplementary Materials of this manuscript.

## Supplementary Materials

The following supporting information can be downloaded at https://www.mdpi.com/article/10.3390/polym16192700/s1, copies of ^1^H NMR, ^13^C NMR, IR, and UV-vis spectra of compounds **2a**–**c**, **3a**–**c**, **5aa**, **5ab**, **5ac**, **5ba**, **5bb**, **5ca**, and **5cb**. Figure S1: UV-Vis spectra of compounds **5aa** (▬), **5ab** (▬), **5ba** (▬), **5bb** (▬), **5ca** (▬), **5cb** (▬), and **5ac** (▬). Figure S2: Particle size distribution histogram determined from SEM image for polymer **5aa**. Figure S3: Particle size distribution histogram determined from SEM image for polymer **5ab**. Figure S4. Particle size distribution histogram determined from SEM image for polymer **5ac**. Figure S5. Particle size distribution histogram determined from SEM image for polymer **5ba**. Figure S6. Particle size distribution histogram determined from SEM image for polymer **5bb**. Figure S7. Particle size distribution histogram determined from SEM image for polymer **5cb**.

## References

[1] Koltzenburg S., Maskos M., Nuyken O. (2017). Polymer Chemistry.

[2] Carraher C.E. (2018). Polymer Chemistry.

[3] Samir A., Ashour F.H., Hakim A.A.A., Bassyouni M. (2022). Recent advances in biodegradable polymers for sustainable applications. npj Mater. Degrad..

[4] Snyder R.L., Fortman D.J., De Hoe D.X., Hillmyer M.A., Dichtel W.R. (2018). Reprocessable acid-degradable polycarbonate vitrimers. Macromolecules.

[5] Zhu Y., Romain C., Williams C.K. (2016). Sustainable polymers from renewable resources. Nature.

[6] Tapper R.J., Longana M.L., Norton A., Potter K.D., Hamerton I. (2020). An Evaluation of Life Cycle Assessment and its Application to the Closed-Loop Recycling of Carbon Fibre Reinforced Polymers. Compos. Part B.

[7] Zhang X., Xiao Z., Liu X., Mei P., Yang Y. (2024). Redox-active polymers as organic electrode materials for sustainable supercapacitors. Renew. Sustain. Energy Rev..

[8] Tour J.M., Kittrell C., Colvin V.L. (2010). Green carbon as a bridge to renewable energy. Nat. Mater..

[9] Bitenc J., Pirnat K., Lužanin O., Dominko R. (2024). Organic Cathodes, a Path toward Future Sustainable Batteries: Mirage or Realistic Future?. Chem. Mater..

[10] Jabeen N., Hussain A., Ullah Z., Haq T.U., Elsharkawy E.M., El-Bahy Z.M., Hessein M.M. (2024). Design and performance evaluation of hierarchical ZnCo2S4@PANI core-shell nanorod arrays for high-efficiency supercapacitor applications. Mater. Chem. Phys..

[11] Hussain A., Jabeen N., Tabassum A., Ali J., Gupta R.K. (2024). 3D-Printed Conducting Polymers for Solid Oxide Fuel Cells. 3D-Printed Conducting Polymers: Fundamentals, Advances, Challenges.

[12] Guin P.S., Das S., Mandal P.C. (2011). Electrochemical Reduction of Quinones in Different Media: A Review. Int. J. Electrochem..

[13] Han C., Li H., Shi R., Zhang T., Tong J., Li J., Li B. (2019). Organic quinones towards advanced electrochemical energy storage: Recent advances and challenges. J. Mater. Chem. A.

[14] Piro B., Reisberg S., Anquetin G., Duc H.-T., Pham M.-C. (2013). Quinone-Based Polymers for Label-Free and Reagentless Electrochemical Immunosensors: Application to Proteins, Antibodies and Pesticides Detection. Biosensors.

[15] Li H., Zhang X., Wang X., Zhang J., Yang Y. (2020). One-pot solvothermal incorporation of graphene into chain-engineered polyquinones for metal-free supercapacitors. Chem. Commun..

[16] Šenica L., Grošelj U., Kasunič M., Kočar D., Stanovnik B., Svete J. (2014). Synthesis of Enaminone-Based Vinylogous Peptides. Eur. J. Org. Chem..

[17] Wang Z., Zhao B., Liu Y., Wan J.-P. (2022). Recent Advances in Reactions Using Enaminone in Water or Aqueous Medium. Adv. Synth. Catal..

[18] Huang J., Yu F. (2021). Recent Advances in Organic Synthesis Based on N,N-Dimethyl Enaminones. Synthesis.

[19] Gaber H.M., Bagley M.C., Muhammad Z.A., Gomha S.M. (2017). Recent developments in chemical reactivity of *N*,*N*-dimethylenamino ketones as synthons for various heterocycles. RSC Adv..

[20] Stanovnik B., Svete J. (2004). Synthesis of Heterocycles from Alkyl 3-(Dimethylamino)propenoates and Related Enaminones. Chem. Rev..

[21] Wang L.-J., Dong P.-J., Zhang G., Zhang F.-M. (2023). Review and Perspectives of β-Keto-enamine-Based Covalent Organic Framework for Photocatalytic Hydrogen Evolution. Energy Fuels.

[22] Edelman P.G., Mathisen R.J., Huang S.J., Culbertson B.M., McGrath J.E. (1985). Poly(amide-enamines). Advances in Polymer Synthesis.

[23] Kimura S. (1968). Preparation of Polyquinolones. Makromol. Chem..

[24] Higashi F., Tai A., Adachi K. (1970). The Reaction Between Diethyl Succinylsuccinate (1,4-Diethoxycarbonyl-2,5-dihydroxy-l,4-cyclohexadiene) and Amines and Its Application to Polymer Synthesis. J. Polym. Sci. Part A-1.

[25] Moore J.A., Kochanowski J.E. (1975). Poly(amine esters) Derived from Diethyl 1,4-Cyclohexanedione-2,5-dicarboxylate. Macromolecules.

[26] Ueda M., Kino K., Hirono T., Imai Y. (1976). Synthesis of Polyenamines by Vinylogous Nucleophilic Substitution Polymerisation of 2,2′-Disubstituted Bis(4-ethoxymethylene-5-Oxazolone) with Diamines. J. Polym. Sci. Polym. Chem. Ed..

[27] Ueda M., Otaira K., Imai Y. (1978). Synthesis of Polyenamines by Vinylogous Nucleophilic Substitution Polymerisation of 1,6-Diethoxy-1,5-hexadiene-3,4-dione with Diamines. J. Polym. Sci. Polym. Chem. Ed..

[28] Ueda M., Funayama M., Imai Y. (1979). Synthesis of Polyenamines with Pendant Hydroxyl Groups by Ring-Opening Polyaddition of 5,5′-Oxalylbis(3,4-dihydro-2*H*-pyran) with Diamines. Polym. J..

[29] Christensen P.R., Schuermann A.M., Loeffler K.E., Helms B.A. (2019). Closed-loop recycling of plastics enabled by dynamic covalent diketoenamine bonds. Nat. Chem..

[30] Deng H., Hu R., Leung A.C.S., Zhao E., Lam J.W.Y., Tang B.Z. (2015). Construction of regio- and stereoregular poly(enaminone)s by multicomponent tandem polymerisations of diynes, diaroyl chloride and primary amines. Polym. Chem..

[31] Deng H., He Z., Lam J.W.Y., Tang B.Z. (2015). Regio- and stereoselective construction of stimuli-responsive macromolecules by a sequential coupling-hydroamination polymerisation route. Polym. Chem..

[32] He B., Zhen S., Wu Y., Hu R., Zhao Z., Qin A., Tang B.Z. (2016). Cu(I)-Catalyzed amino-yne click polymerisation. Polym. Chem..

[33] Imai Y., Sakai N., Sasaki J., Ueda M. (1979). Synthesis of Polyenamines by Vinylogous Nucleophilic Substitution Polymerisation of 1,1′-(3,4-Dioxo-1,5-hexadienylene)di-2-pyrrolidone with Diamines. Makromol. Chem..

[34] Tomažin U., Alič B., Kristl A., Ručigaj A., Grošelj U., Požgan F., Krajnc M., Štefane B., Šebenik U., Svete J. (2018). Synthesis of polyenaminones by acid-catalysed amino–enaminone ‘click’ polymerization. Eur. Polym. J..

[35] Zupanc A., Kotnik T., Štanfel U., Brodnik Žugelj H., Kristl A., Ručigaj A., Matoh L., Pahovnik D., Grošelj U., Opatz T. (2019). Chemical recycling of polyenaminones by transamination reaction via amino–enaminone polymerisation/depolymerization. Eur. Polym. J..

[36] Štanfel U., Kotnik T., Ričko S., Grošelj U., Štefane B., Pirnat K., Žagar E., Genorio B., Svete J. (2022). Synthesis of Optically and Redox Active Polyenaminones from Diamines and α,α′-Bis[(dimethylamino)methylidene]cyclohexanediones. Polymers.

[37] Mujahid M., Yogeeswari P., Sriram D., Basavanag U.M.V., Díaz-Cervantes E., Córdoba-Bahena L., Robles J., Gonnade R.G., Karthikeyan M., Vyas R. (2015). Spirochromone-chalcone conjugates as antitubercular agents: Synthesis, bio evaluation and molecular modeling studies. RSC Adv..

[38] Baker W. (1934). 21. 4: 6- and 2: 4-Diacetylresorcinol. J. Chem. Soc..

[39] Shishmakova T.G., Bardamova M.I., Kotlyarevskii I.L. (1972). Di- and tetraacetylenic amines of resorcinol series. Russ. Chem. Bull..

[40] Assiri M.A., Ali T.E., Ibrahim M.A., El-Amin E.M., Yahia I.S. (2019). 4,6-Diacetylresorcinol in Heterocyclic Synthesis Part II: Synthesis of Some Novel 4,6-Bis(azolyl/azinyl/azepinyl)resorcinols. Heterocycles.

[41] Bensalah, Gadri, Cañizares P., Sáez C., Lobato J., Rodrigo M.A. (2005). Electrochemical Oxidation of Hydroquinone, Resorcinol, and Catechol on Boron-Doped Diamond Anodes. Environ. Sci. Technol..

[42] Pirnat K., Bitenc J., Vizintin A., Krajnc A., Tcherychova E. (2018). Indirect Synthesis Route toward Cross-Coupled Polymers for High Voltage Organic Positive Electrodes. Chem. Mater..

[43] Liang K., Li X., Wei D., Jin C., Liu C., Xia C. (2023). Deprotection of benzyl-derived groups via photochemically mesolytic cleavage of C–N and C–O bonds. Chem.

[44] Auger C., Gohy J.-F., Poizot P., Vlad A. (2019). A H-bond stabilized quinone electrode material for Li–organic batteries: The strength of weak bonds. Chem. Sci..

[45] Bitenc J., Lindahl N., Vizintin A., Abdelhamid M.E., Dominko R., Johansson P. (2020). Concept and electrochemical mechanism of an Al metal anode–organic cathode battery. Energy Storage Mater..

[46] Narayan R., Blagojević A., Mali G., Vélez Santa J.F., Bitenc J., Randon-Vitanova A., Dominko R. (2022). Nanostructured Poly(hydroquinonyl-benzoquinonyl sulfide)/Multiwalled Carbon Nanotube Composite Cathodes: Improved Synthesis and Performance for Rechargeable Li and Mg Organic Batteries. Chem. Mater..

