# Supplementary material for: Synthesis and Redox Activity of Polyenaminones for Sustainable Energy Storage Applications
Source: Polymers (Basel). 2024 Sep 24;16(19):2700. doi: 10.3390/polym16192700 (PMC11478993; doi:10.3390/polym16192700)
Supplement: Supplementary file 1 [file polymers-16-02700-s001.zip › polymers-3144151-supplementary.pdf]

polymers

Supporting Information for the Article

Synthesis and redox activity of polyenaminones for sustainable energy storage applications

Tomaž Kotnik [1], Svit Menart [1,2], Žan Adam [1,2], Jan Bitenc [2], Luka Ciber [1], Uroš Grošelj [1], Nejc Petek [1], Bogdan Štefane [1], Jurij Svete [1*], and Boštjan Genorio [1*]

[1] University of Ljubljana, Faculty of Chemistry and Chemical Technology, Večna pot 113, 1000 Ljubljana, Slovenia; info@fkkt.uni-lj.si
[2] National Institute of Chemistry, Hajdrihova 19, 1000 Ljubljana, Slovenia; glavna.pisarna@ki.si
* Correspondence: bostjan.genorio@fkkt.uni-lj.si; Tel.: +386 1 479 8586 (BG) and jurij.svete@fkkt.uni-lj.si; Tel.: +386 1 479 8562 (JS).

Table of contents

1. Copies of NMR spectra of compounds **2**, **3**, and **5** S2
2. Copies of IR spectra of compounds **2**, **3**, and **5** S15
3. Copies of UV spectra of compounds **2**, **3**, and **5** S20
4. Particle size distribution histograms determined from SEM images for polymers **5aa, 5ab, 5ac, 5ba, 5bb, and 5cb** S24

1. Copies of ^{1}H NMR and ^{13}C NMR spectra of compounds 2, 3 and 5.

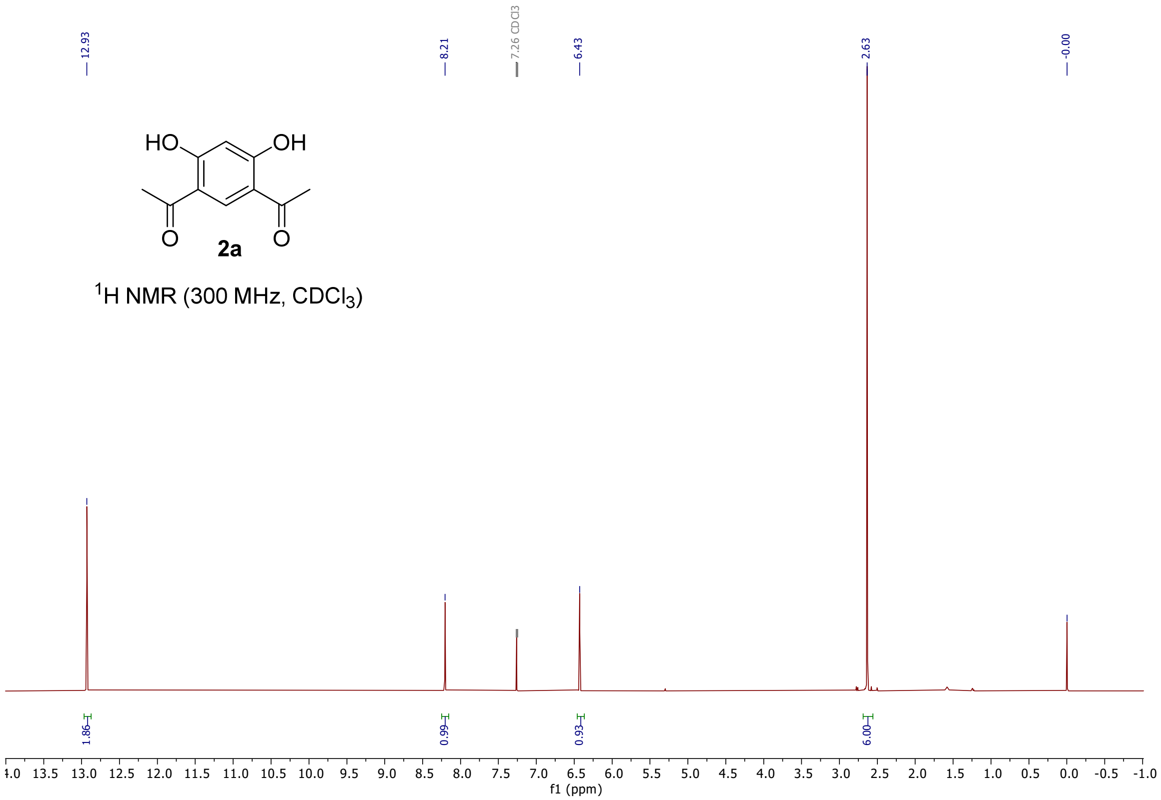

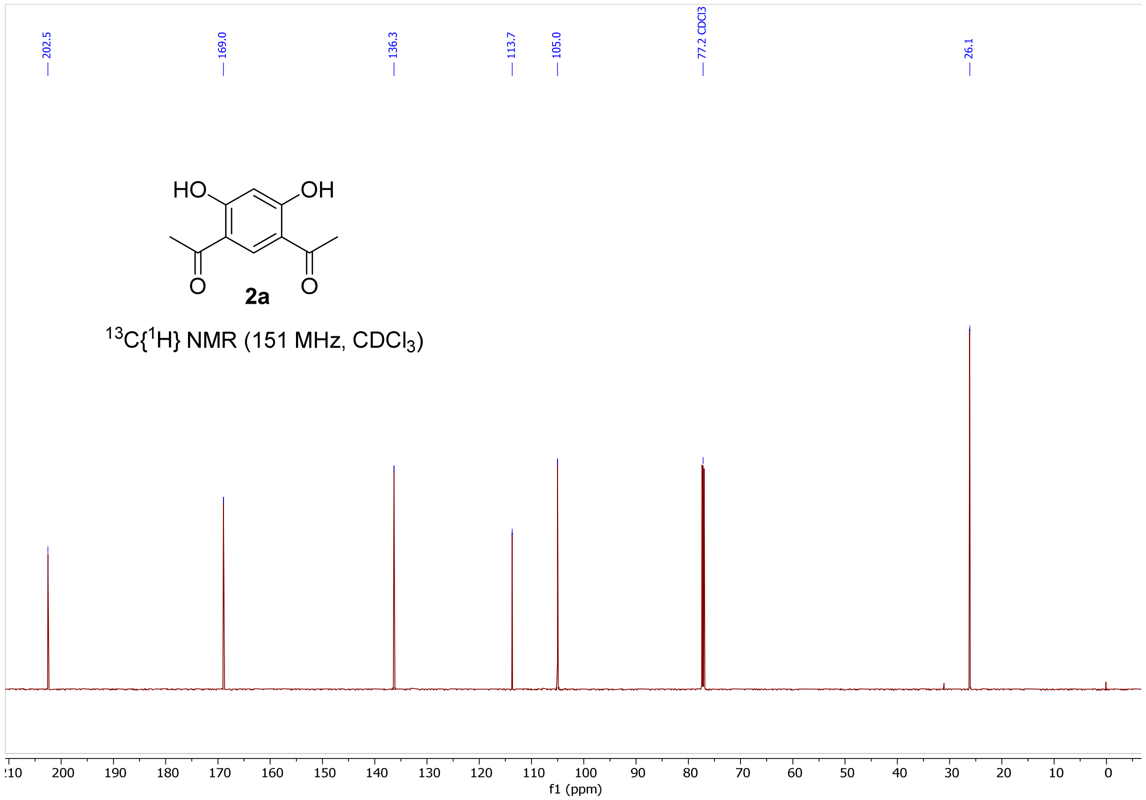

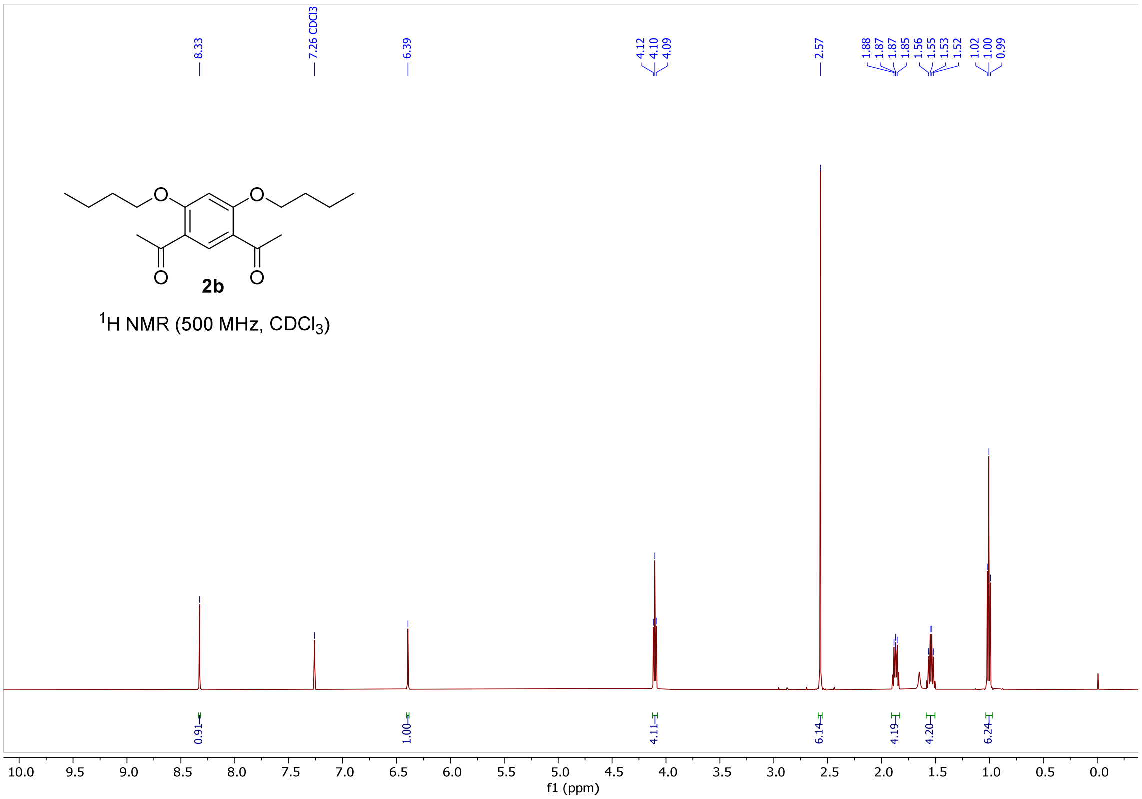
2b
^{1}H NMR (500 MHz, $CDCl_3$)
8.33
7.26 CDCl3
6.39
4.12
4.10
4.09
2.57
1.88
1.87
1.87
1.85
1.56
1.55
1.53
1.52
1.02
1.00
0.99
0.91
1.00
4.11
6.14
4.19
4.20
6.24
f1 (ppm)

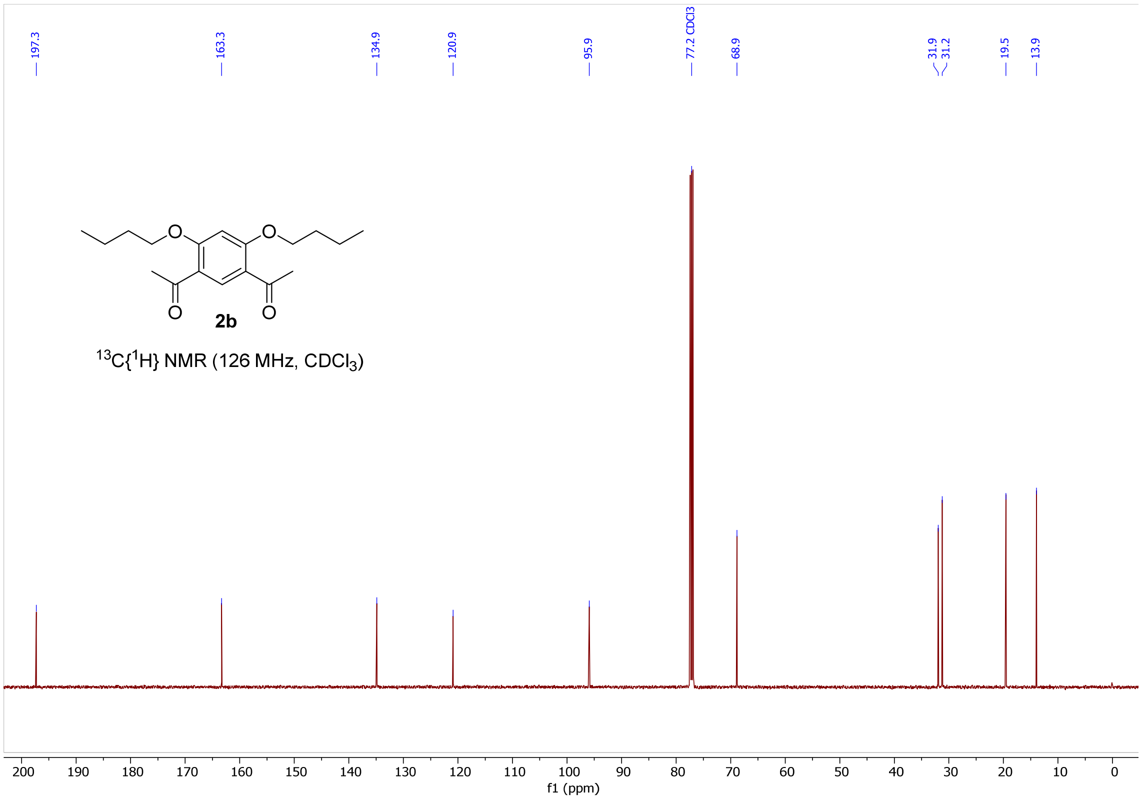
2b
^{13}C{^{1}H} NMR (126 MHz, $CDCl_3$)
197.3
163.3
134.9
120.9
95.9
77.2 CDCl3
68.9
31.9
31.2
19.5
13.9
f1 (ppm)

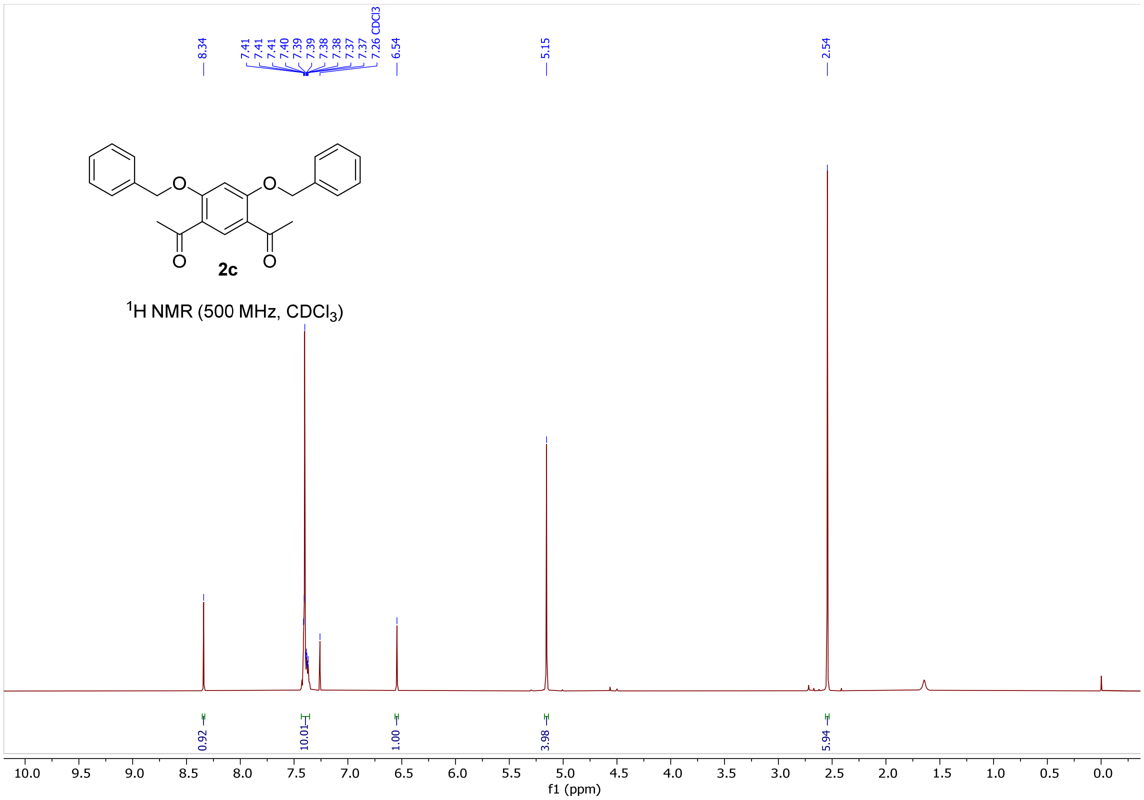
8.34
7.41
7.41
7.41
7.40
7.39
7.39
7.38
7.38
7.37
7.37
7.26 CDCl3
6.54
5.15
2.54
2c
¹H NMR (500 MHz, CDCl₃)
0.92
10.01
1.00
3.98
5.94
10.0 9.5 9.0 8.5 8.0 7.5 7.0 6.5 6.0 5.5 5.0 4.5 4.0 3.5 3.0 2.5 2.0 1.5 1.0 0.5 0.0
f1 (ppm)

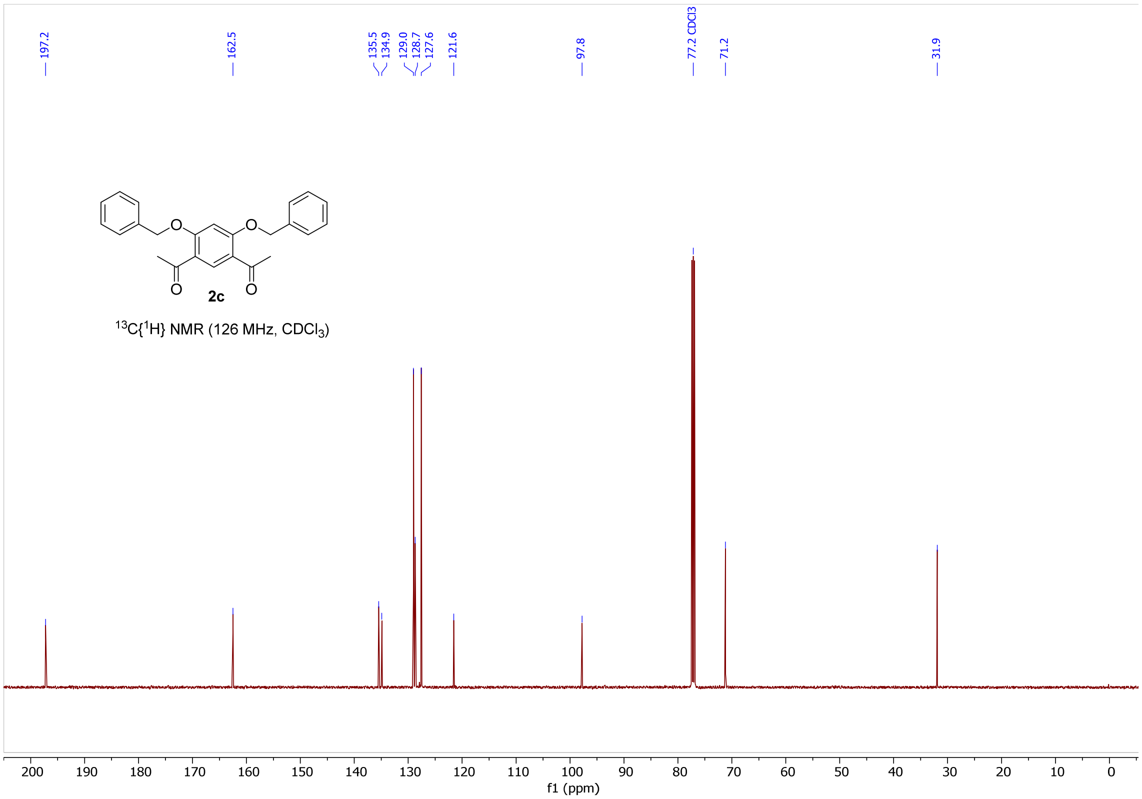
197.2
162.5
135.5
134.9
129.0
128.7
127.6
121.6
97.8
77.2 CDCl3
71.2
31.9
2c
¹³C{¹H} NMR (126 MHz, CDCl₃)
200 190 180 170 160 150 140 130 120 110 100 90 80 70 60 50 40 30 20 10 0
f1 (ppm)

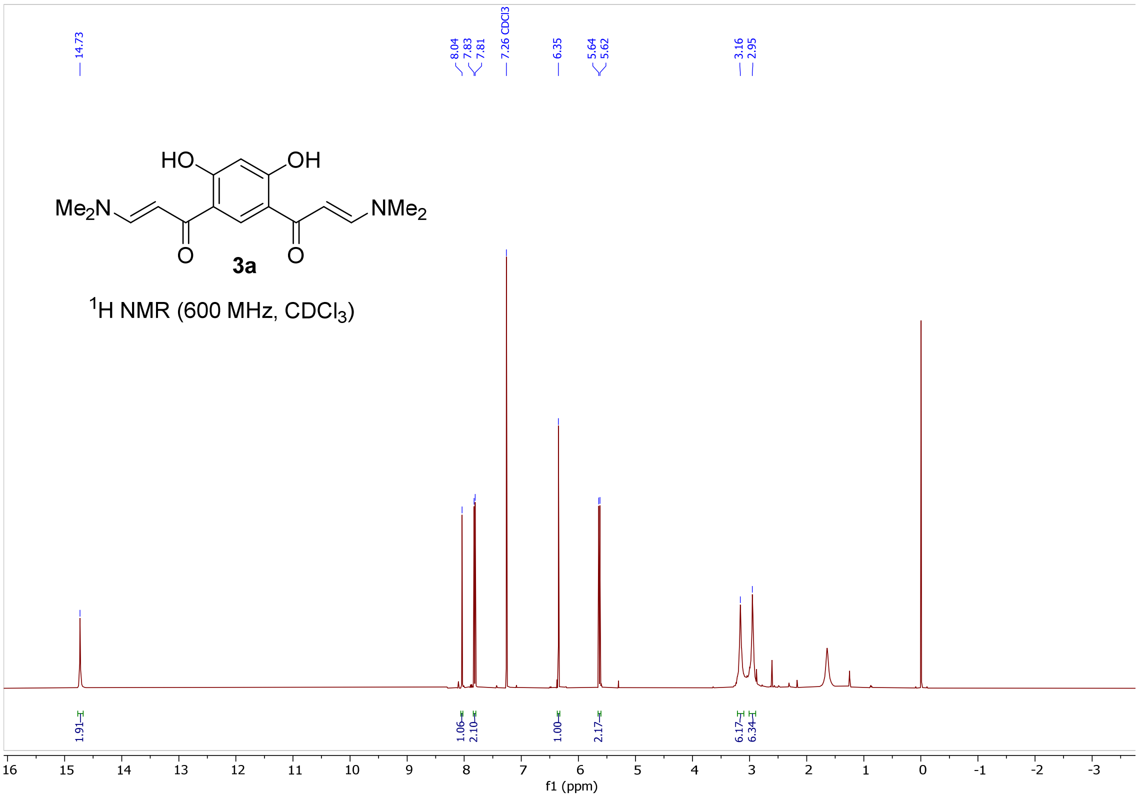
14.73
8.04
7.83
7.81
7.26 CDCl3
6.35
5.64
5.62
3.16
2.95
HO
OH
Me2N
NMe2
O
3a
O
1H NMR (600 MHz, CDCl3)
1.91
1.06
2.10
1.00
2.17
6.17
6.34
16 15 14 13 12 11 10 9 8 7 6 5 4 3 2 1 0 -1 -2 -3
f1 (ppm)

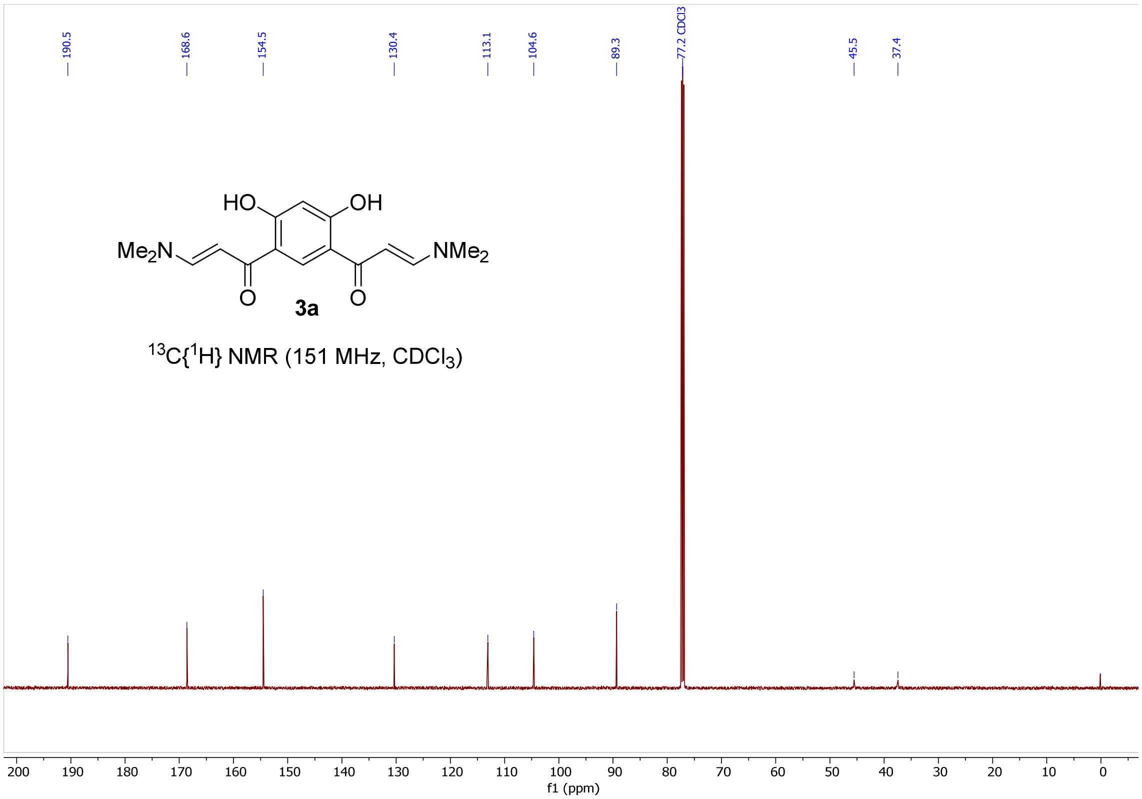
190.5
168.6
154.5
130.4
113.1
104.6
89.3
77.2 CDCl3
45.5
37.4
HO
OH
Me2N
NMe2
O
3a
O
13C{1H} NMR (151 MHz, CDCl3)
200 190 180 170 160 150 140 130 120 110 100 90 80 70 60 50 40 30 20 10 0
f1 (ppm)

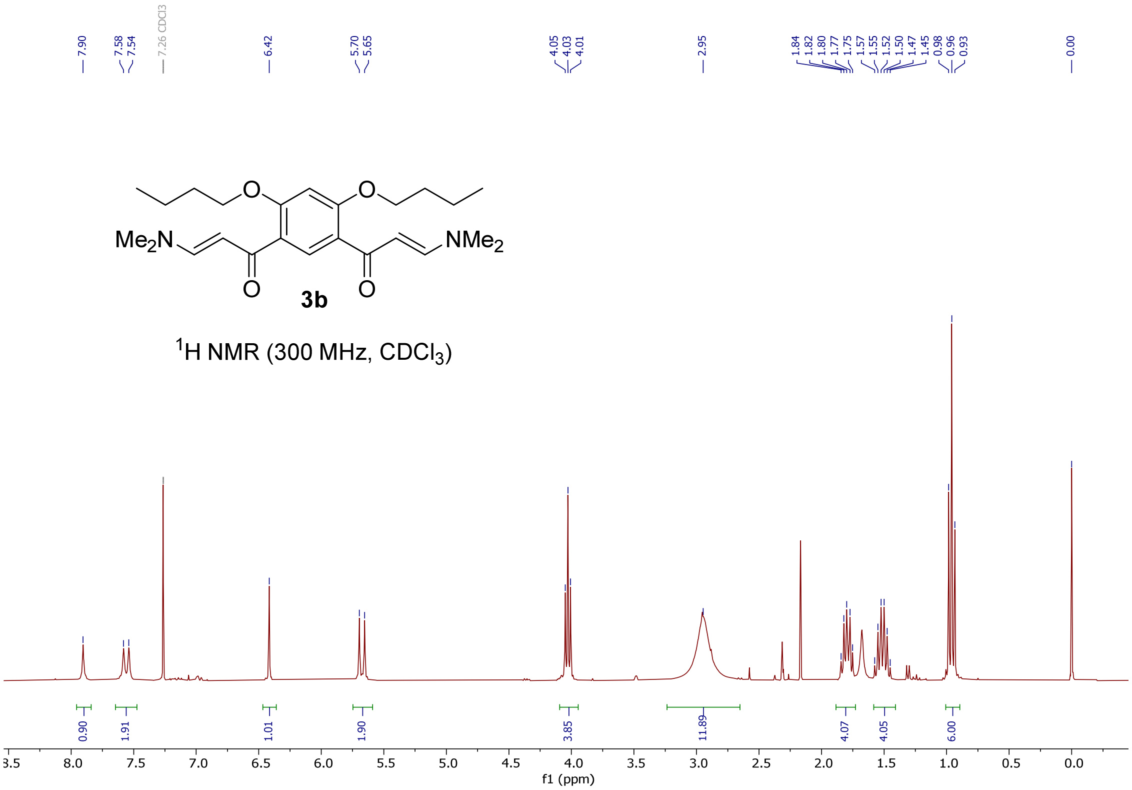
7.90
7.58
7.54
7.26 CDCl3
6.42
5.70
5.65
4.05
4.03
4.01
2.95
1.84
1.82
1.80
1.77
1.75
1.57
1.55
1.52
1.50
1.47
1.45
0.98
0.96
0.93
0.00
Me2N
NMe2
O
3b
1H NMR (300 MHz, CDCl3)
0.90
1.91
1.01
1.90
3.85
11.89
4.07
4.05
6.00
f1 (ppm)

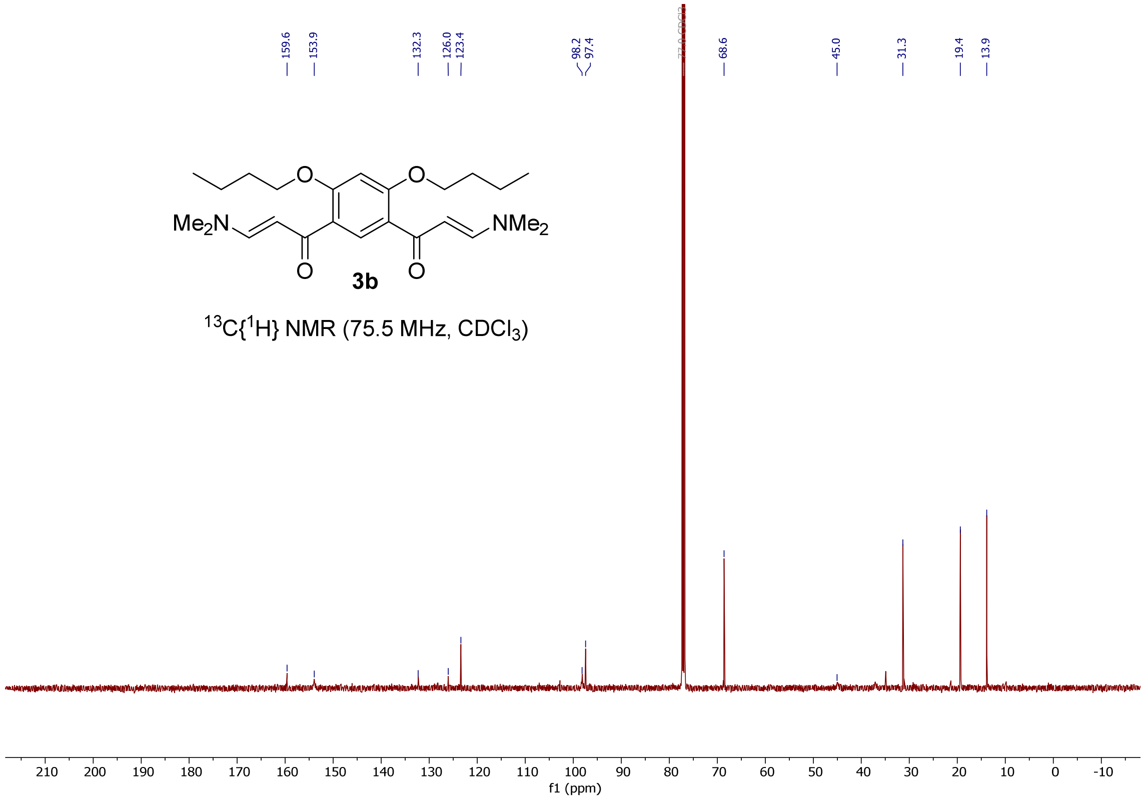
159.6
153.9
132.3
126.0
123.4
98.2
97.4
68.6
45.0
31.3
19.4
13.9
Me2N
NMe2
O
3b
13C{1H} NMR (75.5 MHz, CDCl3)
f1 (ppm)

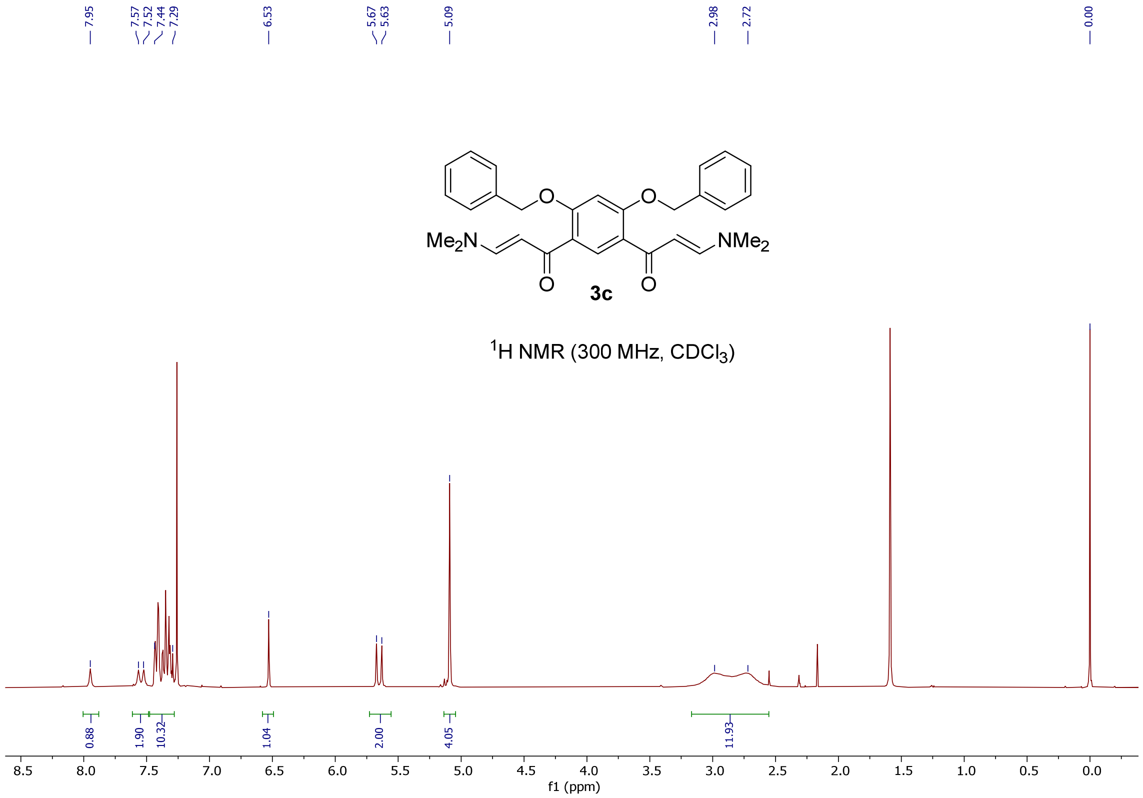
7.95
7.57
7.52
7.44
7.29
6.53
5.67
5.63
5.09
2.98
2.72
0.00
Me2N
NMe2
O
3c
1H NMR (300 MHz, CDCl3)
0.88
1.90
10.32
1.04
2.00
4.05
11.93
f1 (ppm)

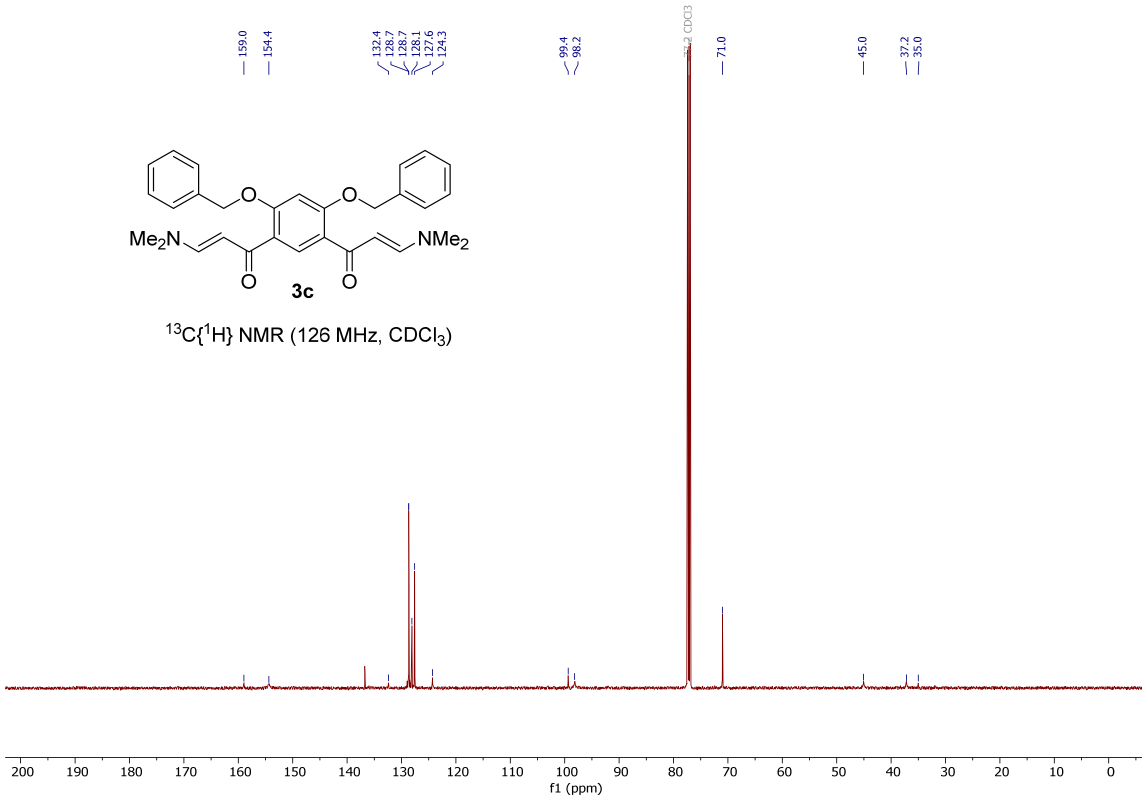
159.0
154.4
132.4
128.7
128.7
128.1
127.6
124.3
99.4
98.2
77.2 CDCl3
71.0
45.0
37.2
35.0
Me2N
NMe2
O
3c
13C{1H} NMR (126 MHz, CDCl3)
f1 (ppm)

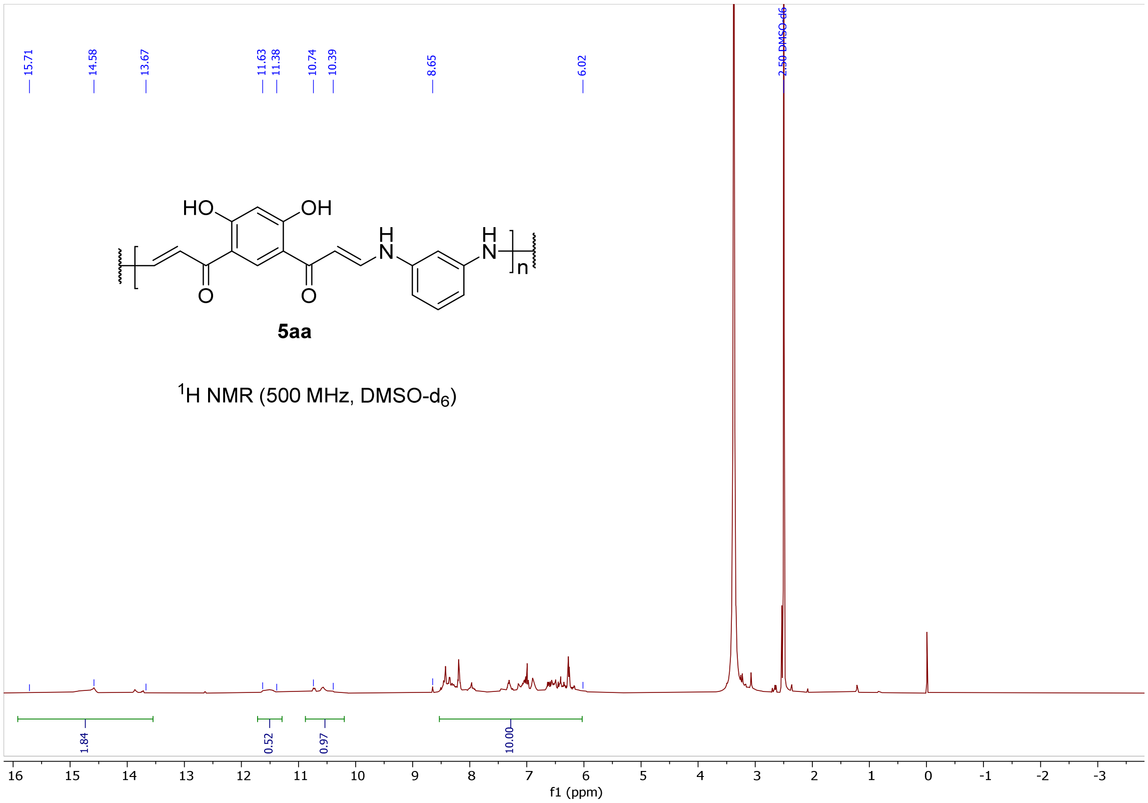
5aa
1H NMR (500 MHz, DMSO-d6)
f1 (ppm)

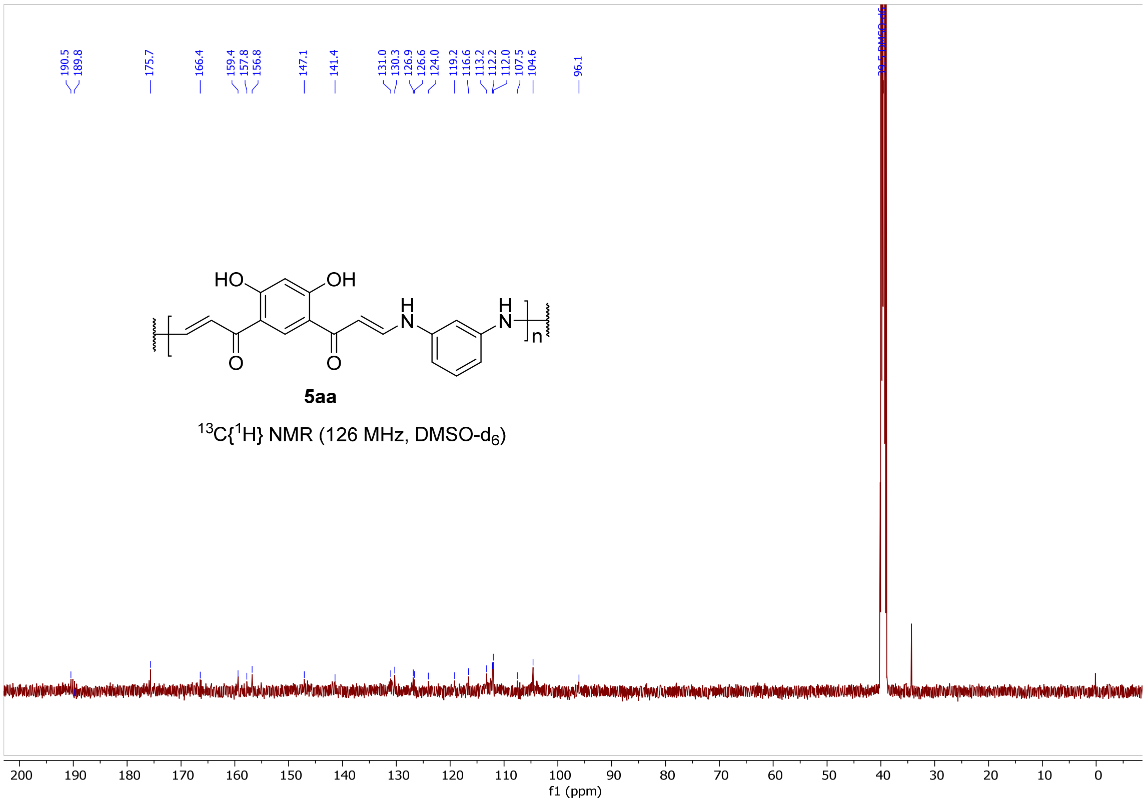
5aa
13C{1H} NMR (126 MHz, DMSO-d6)
f1 (ppm)

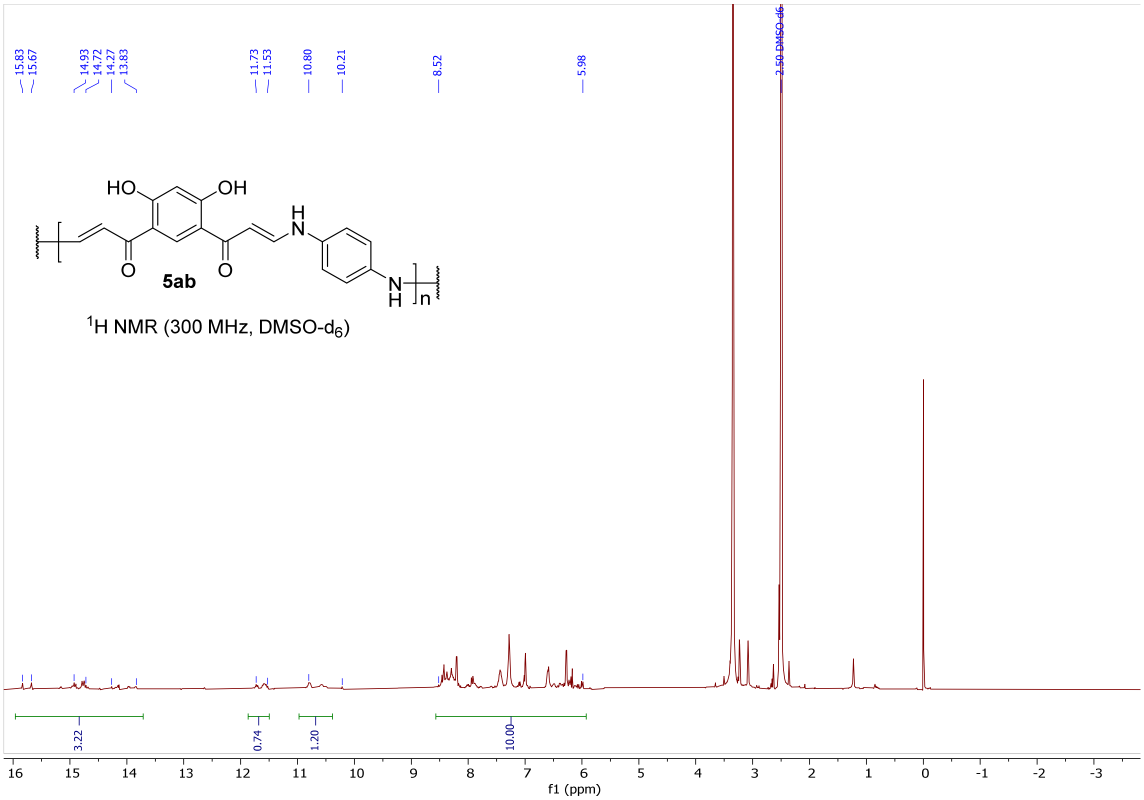
15.83
15.67
14.93
14.72
14.27
13.83
11.73
11.53
10.80
10.21
8.52
5.98
2.50 DMSO-d6
HO
OH
H
N
O
5ab
O
N
H
n
^{1}H NMR (300 MHz, DMSO-d$_6$)
3.22
0.74
1.20
10.00
f1 (ppm)

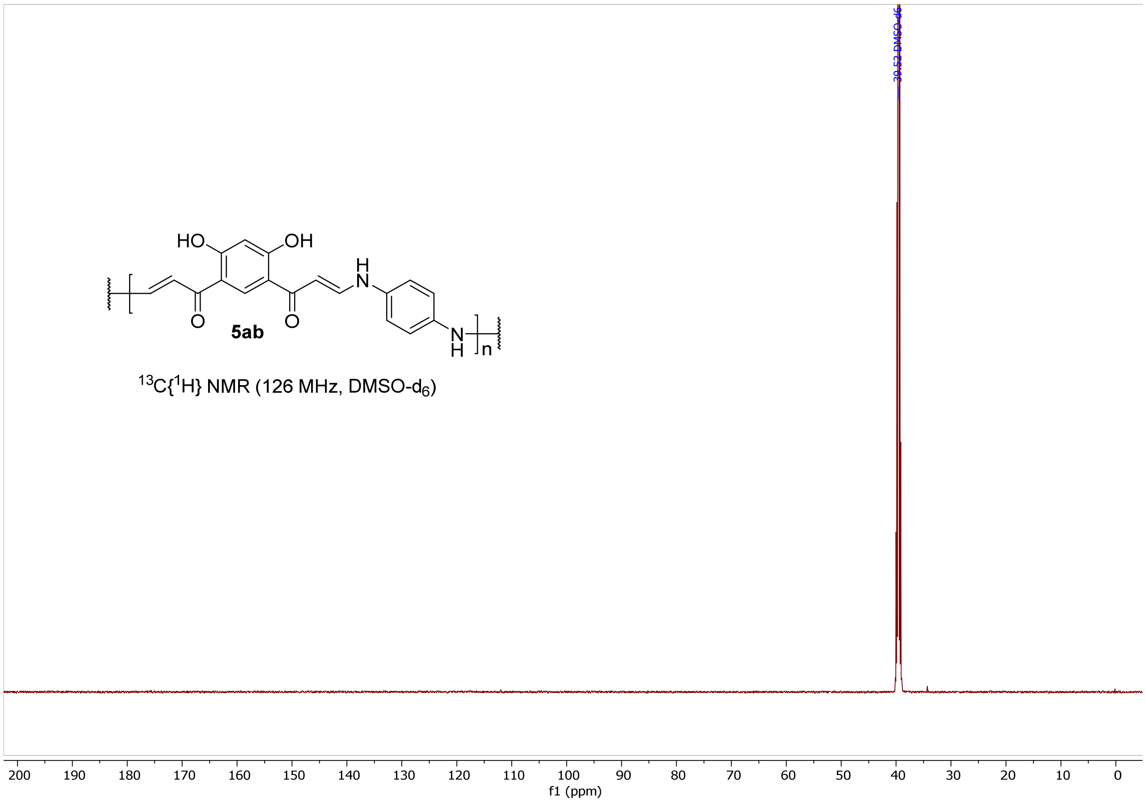
39.52 DMSO-d6
HO
OH
H
N
O
5ab
O
N
H
n
^{13}C{^{1}H} NMR (126 MHz, DMSO-d$_6$)
f1 (ppm)

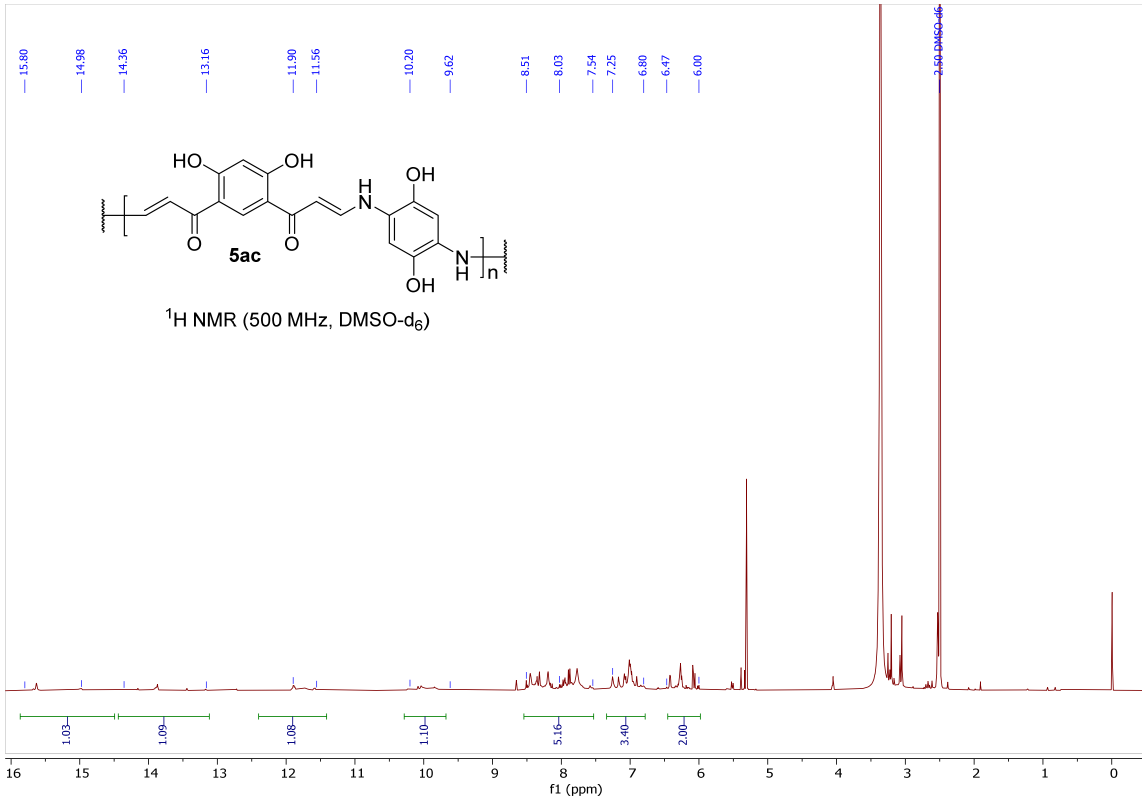
15.80
14.98
14.36
13.16
11.90
11.56
10.20
9.62
8.51
8.03
7.54
7.25
6.80
6.47
6.00
2.50 DMSO-d6
HO
OH
OH
H
N
5ac
O
O
N
H
n
OH
1H NMR (500 MHz, DMSO-d6)
1.03
1.09
1.08
1.10
5.16
3.40
2.00
f1 (ppm)

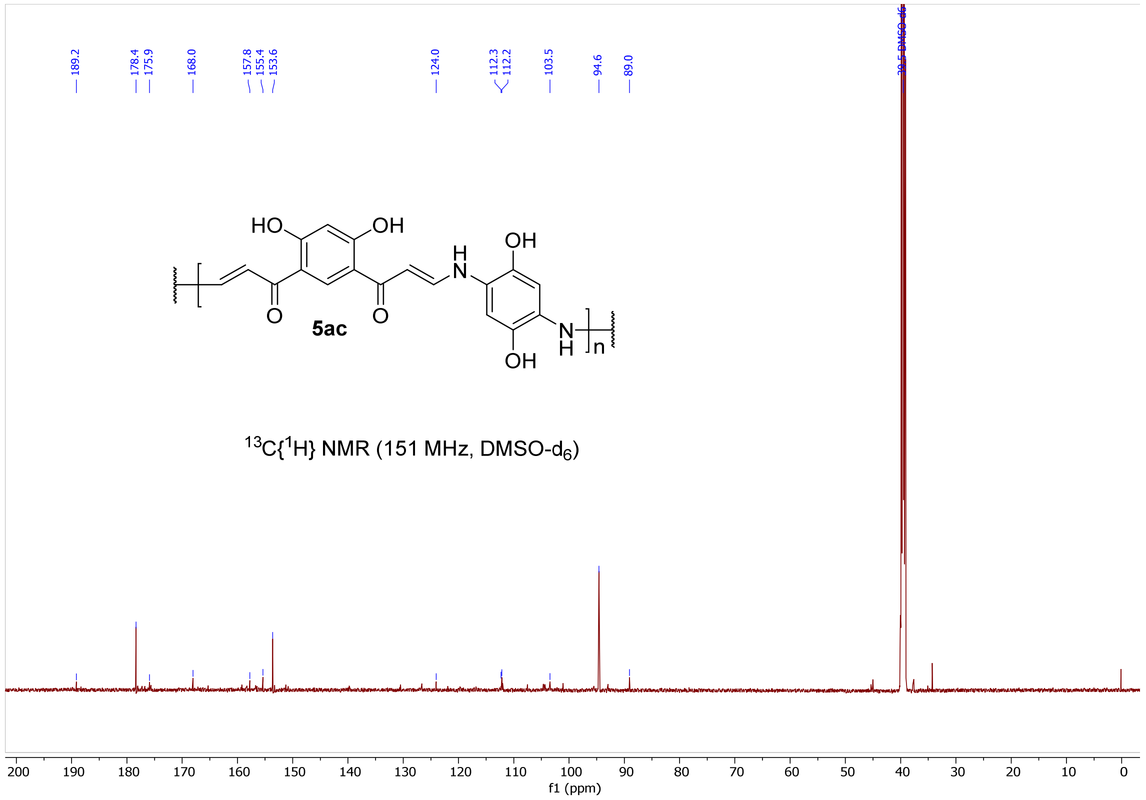
189.2
178.4
175.9
168.0
157.8
155.4
153.6
124.0
112.3
112.2
103.5
94.6
89.0
HO
OH
OH
H
N
5ac
O
O
N
H
n
OH
13C{1H} NMR (151 MHz, DMSO-d6)
f1 (ppm)

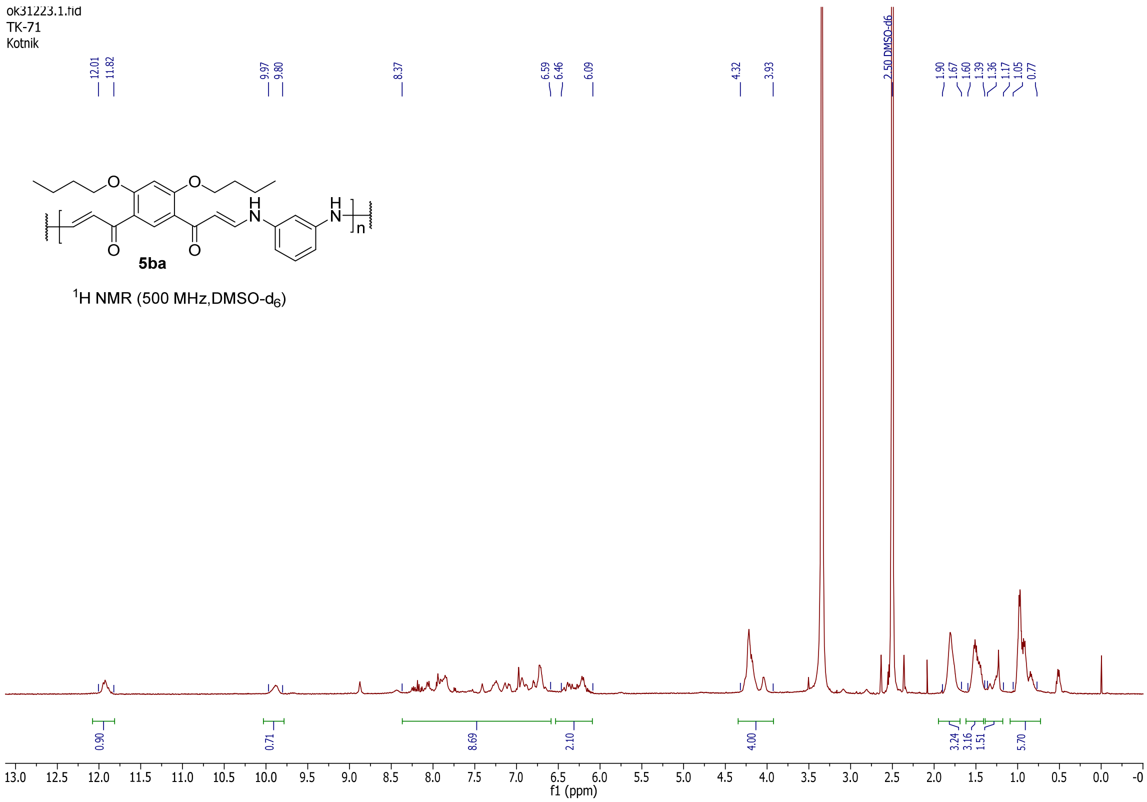
ok31223.1.fid
TK-71
Kotnik
5ba
^{1}H NMR (500 MHz,DMSO-d$_6$)
f1 (ppm)

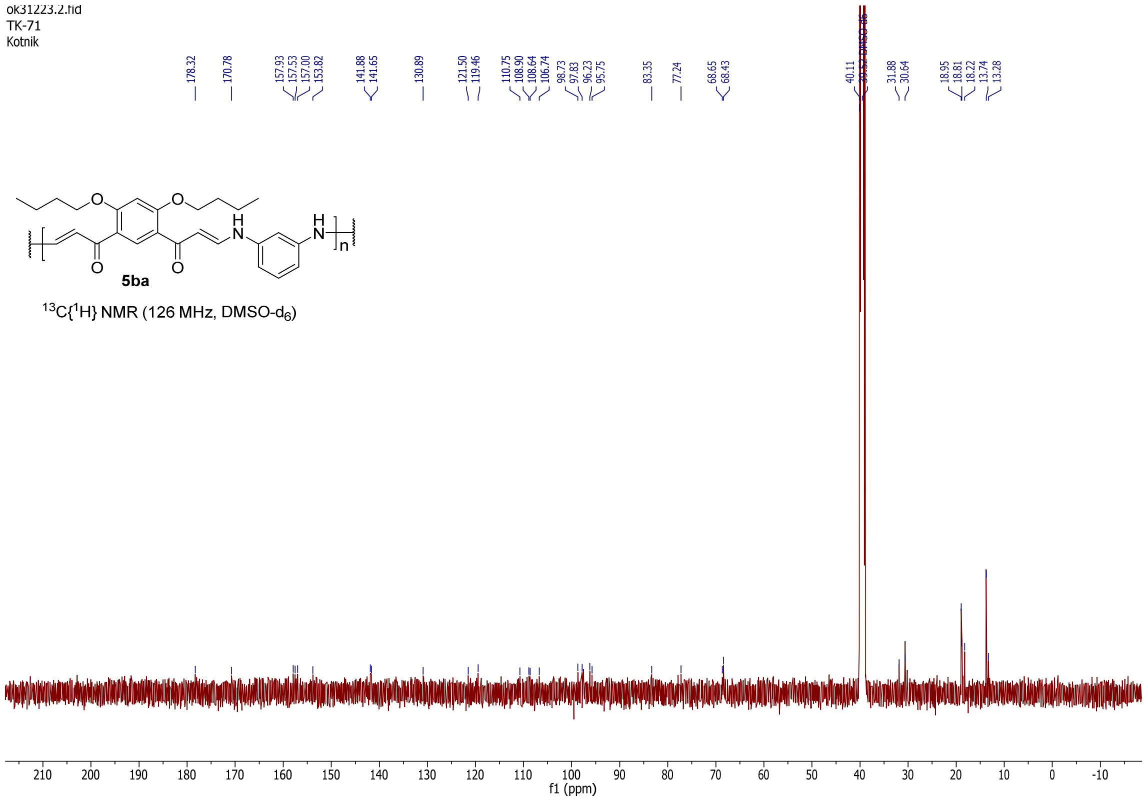
ok31223.2.fid
TK-71
Kotnik
5ba
^{13}C{^{1}H} NMR (126 MHz, DMSO-d$_6$)
f1 (ppm)

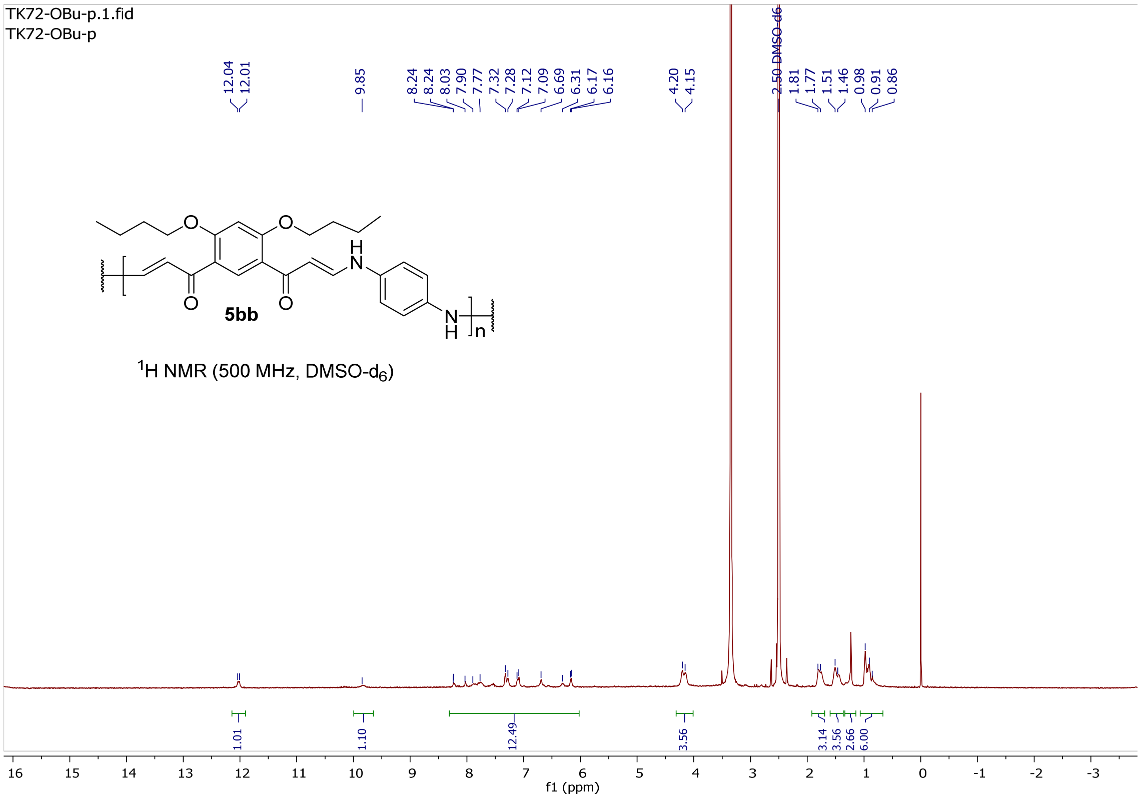
TK72-OBu-p.1.fid
TK72-OBu-p
12.04
12.01
9.85
8.24
8.24
8.03
7.90
7.77
7.32
7.28
7.12
7.09
6.69
6.31
6.17
6.16
4.20
4.15
2.50 DMSO-d6
1.81
1.77
1.51
1.46
0.98
0.91
0.86
5bb
1H NMR (500 MHz, DMSO-d6)
1.01
1.10
12.49
3.56
3.14
3.56
2.66
6.00
f1 (ppm)

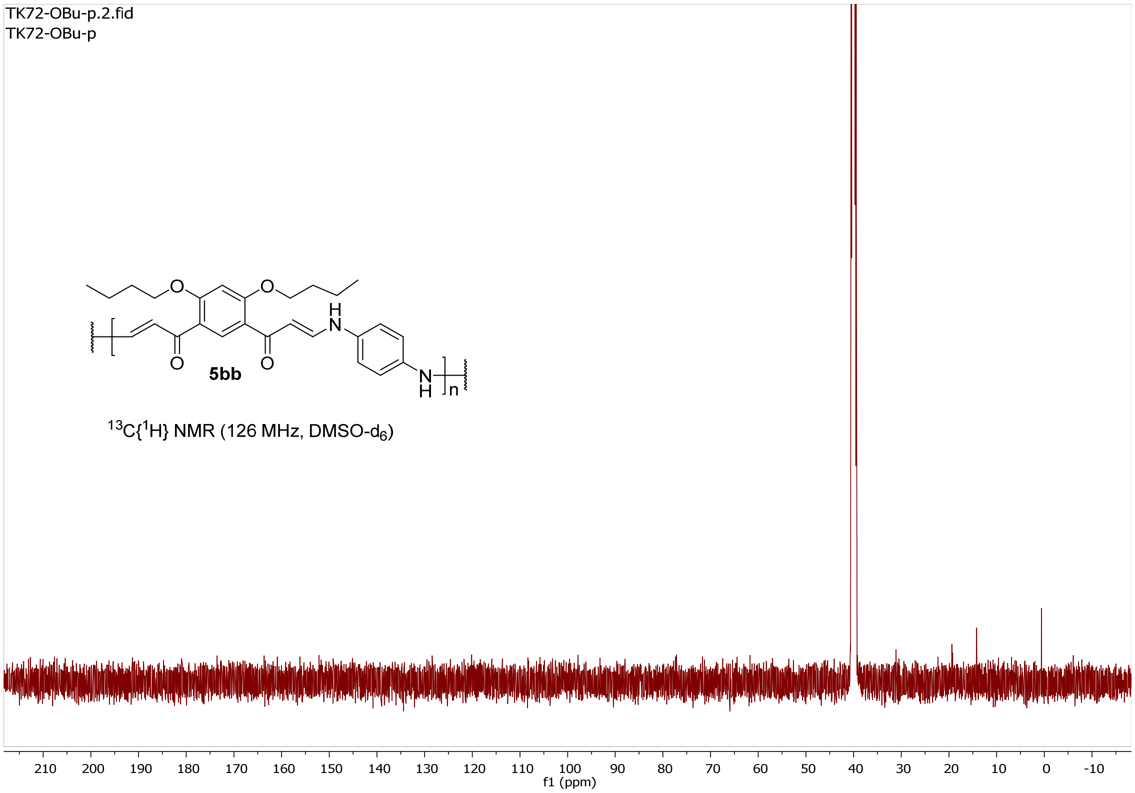
TK72-OBu-p.2.fid
TK72-OBu-p
5bb
13C{1H} NMR (126 MHz, DMSO-d6)
f1 (ppm)

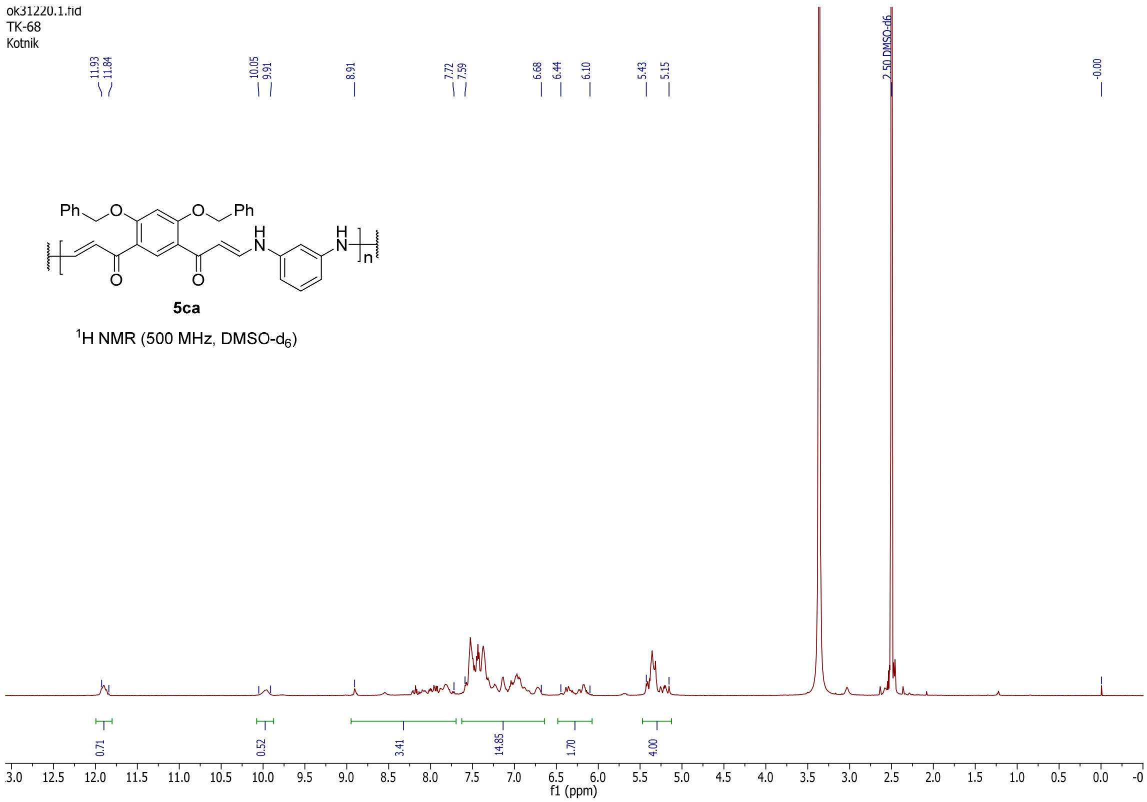
ok31220.1.fid
TK-68
Kotnik
5ca
^{1}H NMR (500 MHz, DMSO-d$_6$)
2.50 DMSO-d6
f1 (ppm)

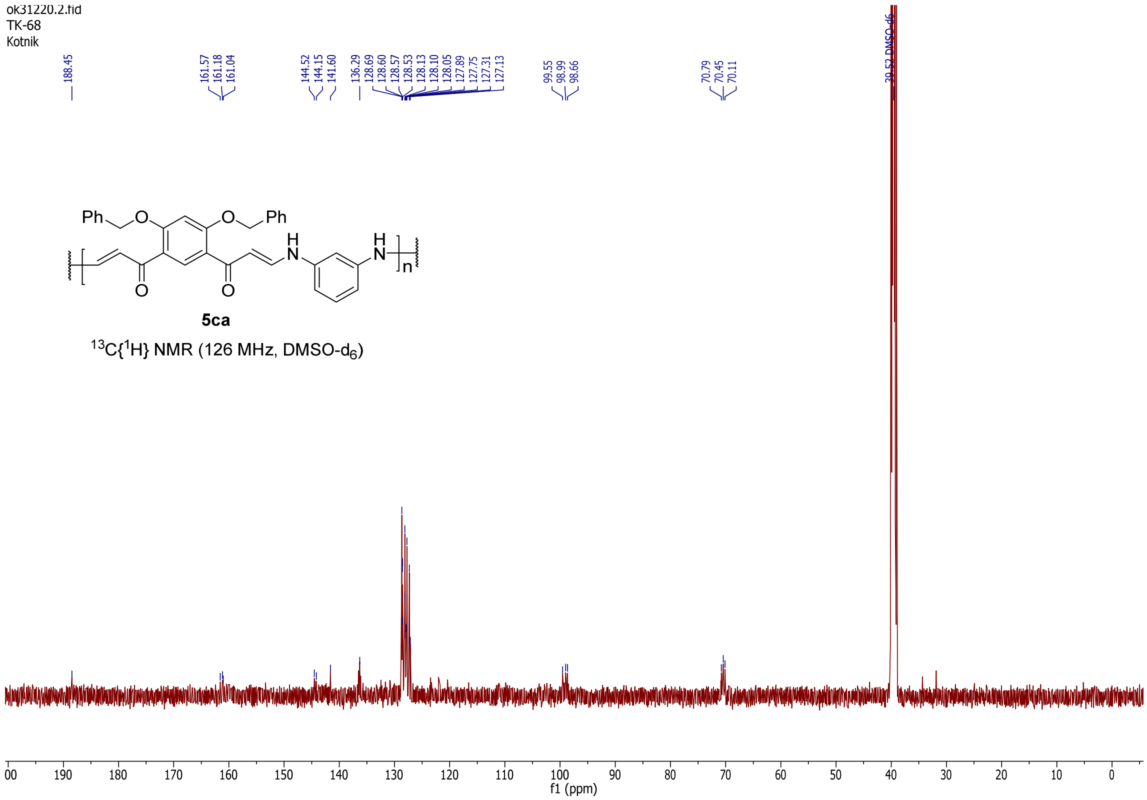
ok31220.2.fid
TK-68
Kotnik
5ca
^{13}C{^{1}H} NMR (126 MHz, DMSO-d$_6$)
39.52 DMSO-d6
f1 (ppm)

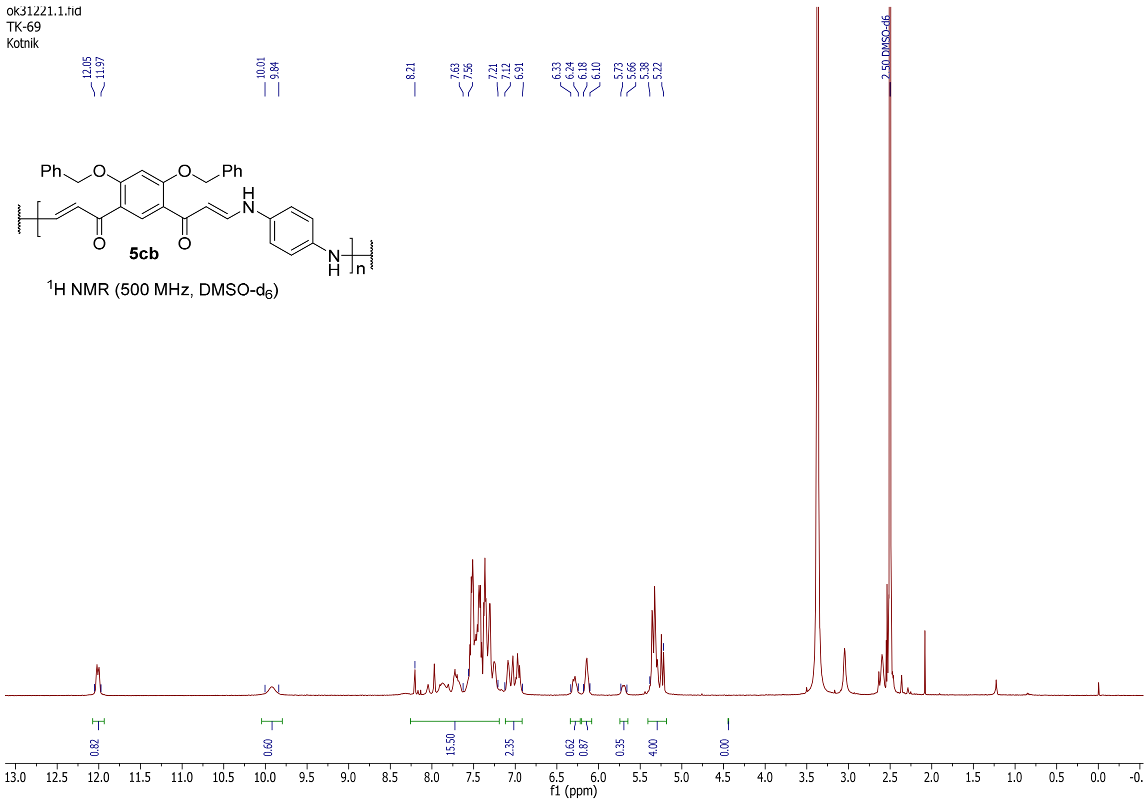
ok31221.1.fid
TK-69
Kotnik
12.05 11.97 10.01 9.84 8.21 7.63 7.56 7.21 7.12 6.91 6.33 6.24 6.18 6.10 5.73 5.66 5.38 5.22
2.50 DMSO-d6
Ph O O Ph
H N
O O
5cb
N H n
1H NMR (500 MHz, DMSO-d6)
0.82 0.60 15.50 2.35 0.62 0.87 0.35 4.00 0.00
f1 (ppm)

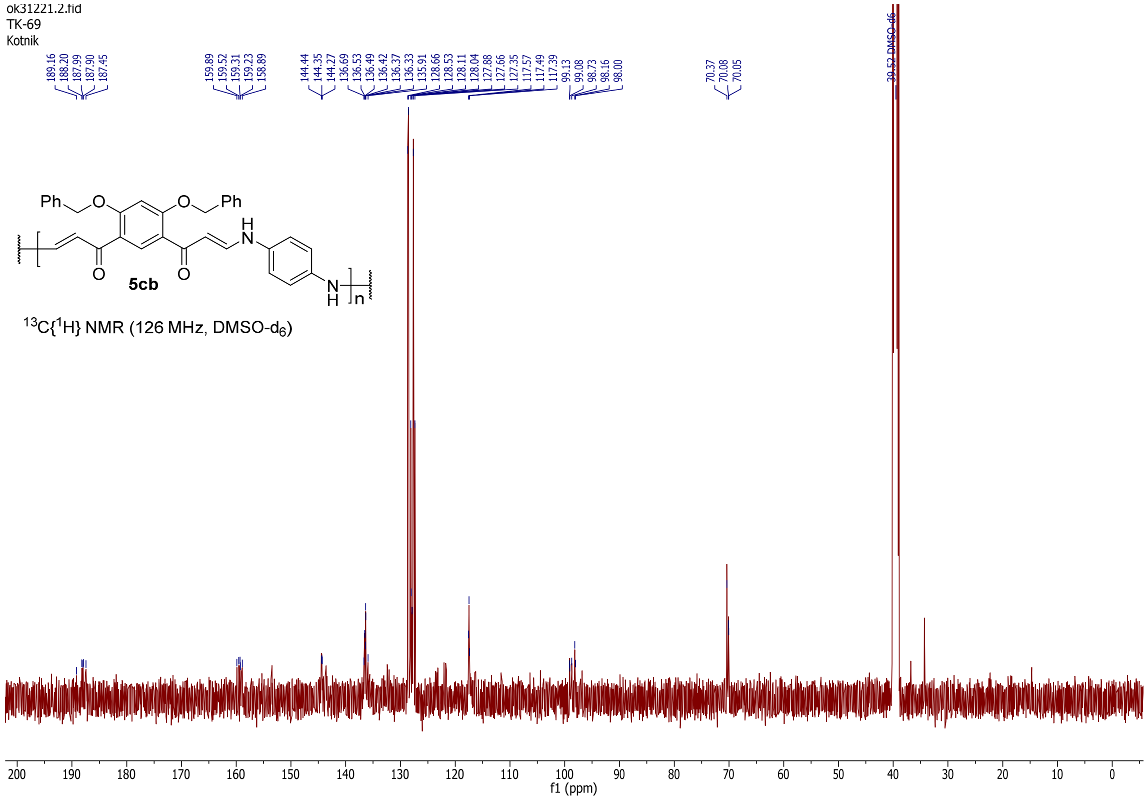
ok31221.2.fid
TK-69
Kotnik
189.16 188.20 187.99 187.90 187.45
159.89 159.52 159.31 159.23 158.89
144.44 144.35 144.27 136.69 136.53 136.49 136.42 136.37 136.33 135.91 128.66 128.53 128.11 128.04 127.88 127.66 127.35 117.57 117.49 117.39 99.13 99.08 98.73 98.16 98.00
70.37 70.08 70.05
39.52 DMSO-d6
Ph O O Ph
H N
O O
5cb
N H n
13C{1H} NMR (126 MHz, DMSO-d6)
f1 (ppm)

2. Copies of IR spectra of compounds 2, 3, and 5.

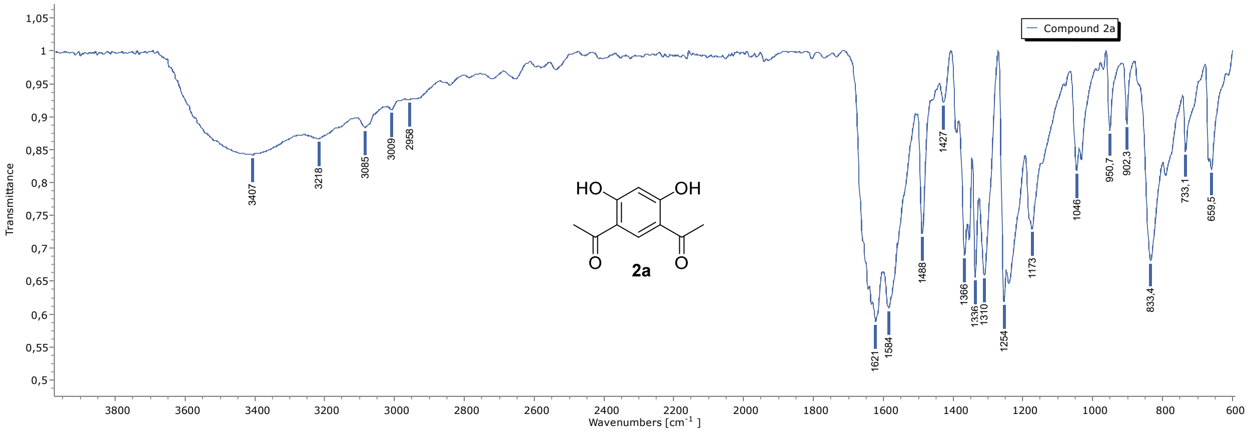

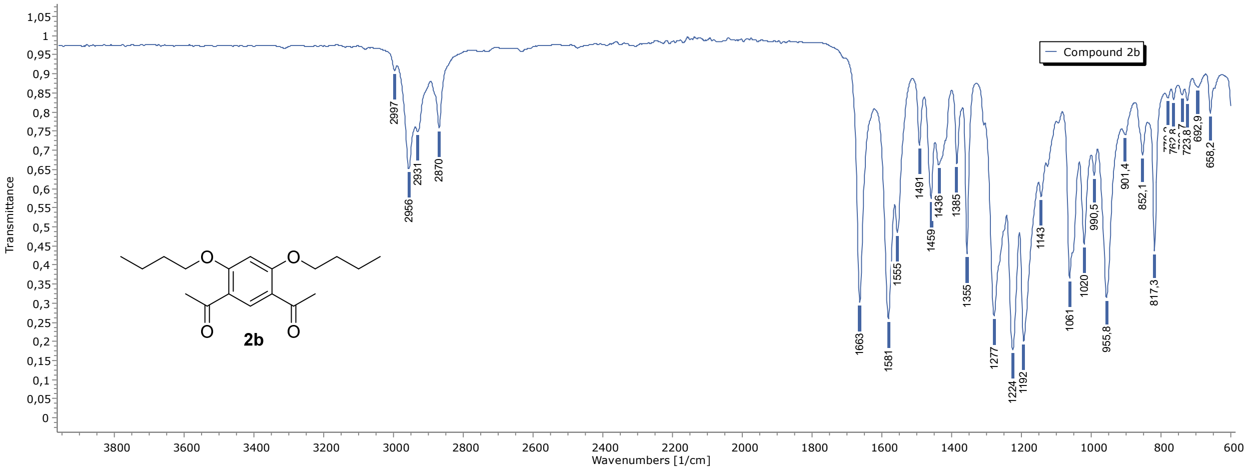

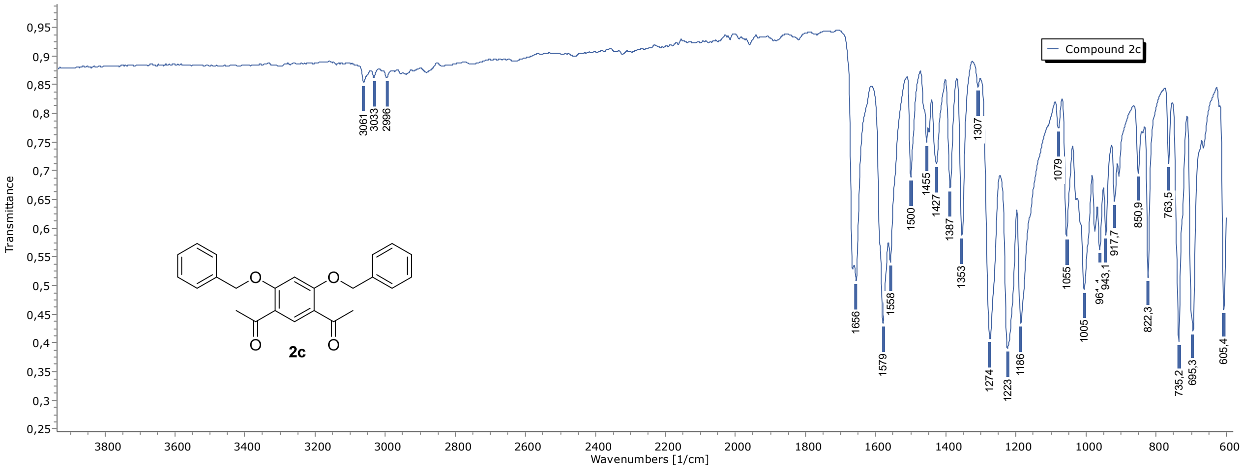

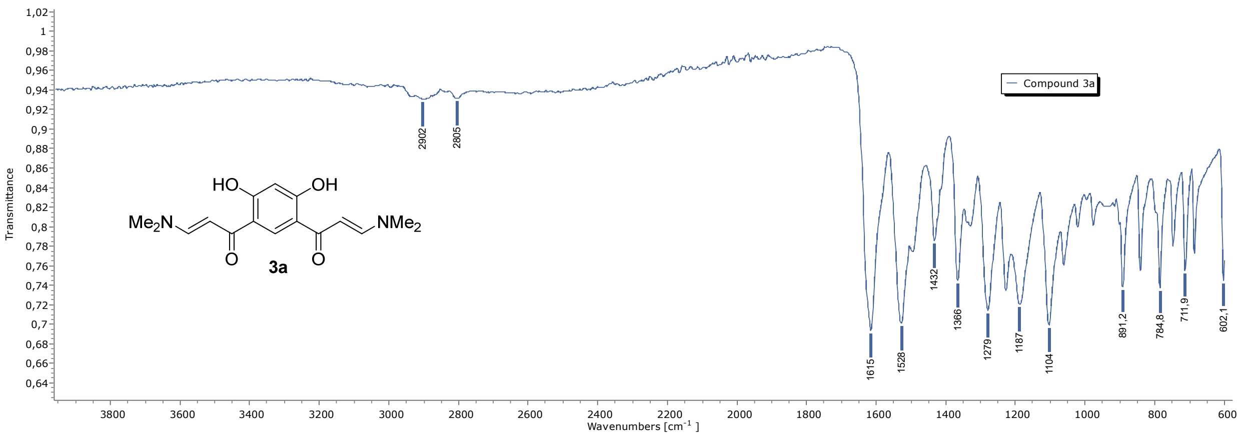
Compound 3a
Transmittance
Wavenumbers [cm-1]
2902
2805
1615
1528
1432
1366
1279
1187
1104
891,2
784,8
711,9
602,1
HO
OH
Me2N
NMe2
O
O
3a

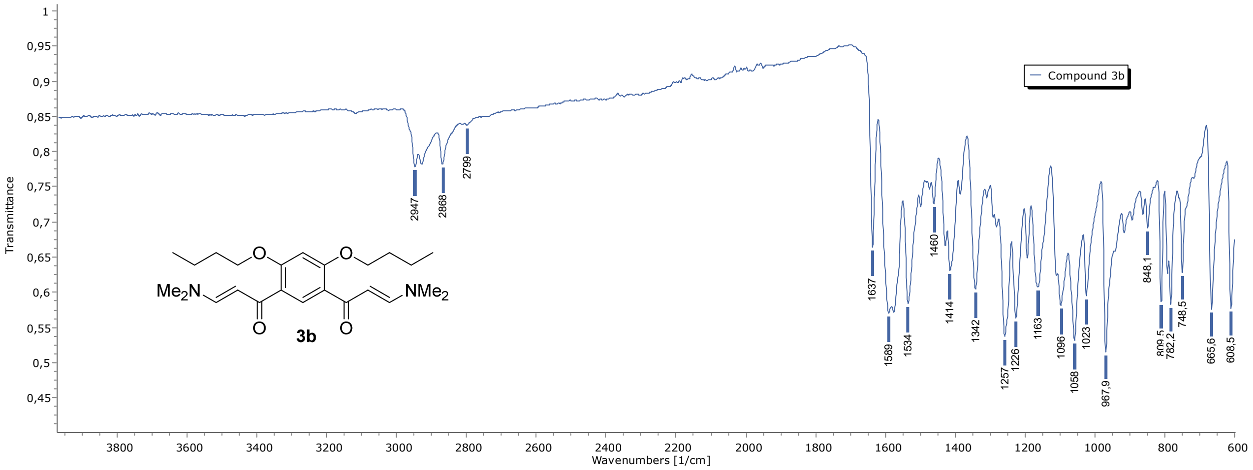
Compound 3b
Transmittance
Wavenumbers [1/cm]
2947
2868
2799
1637
1589
1534
1460
1414
1342
1257
1226
1163
1096
1058
1023
967,9
848,1
809,5
782,2
748,5
665,6
608,5
O
O
Me2N
NMe2
O
O
3b

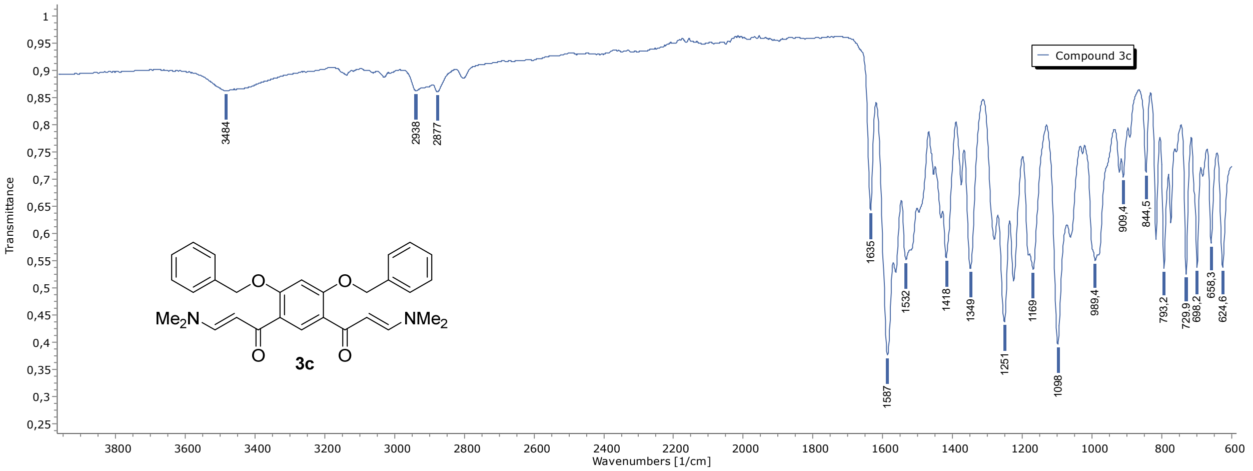
Compound 3c
Transmittance
Wavenumbers [1/cm]
3484
2938
2877
1635
1587
1532
1418
1349
1251
1169
1098
989,4
909,4
844,5
793,2
729,9
698,2
658,3
624,6
O
O
Me2N
NMe2
O
O
3c

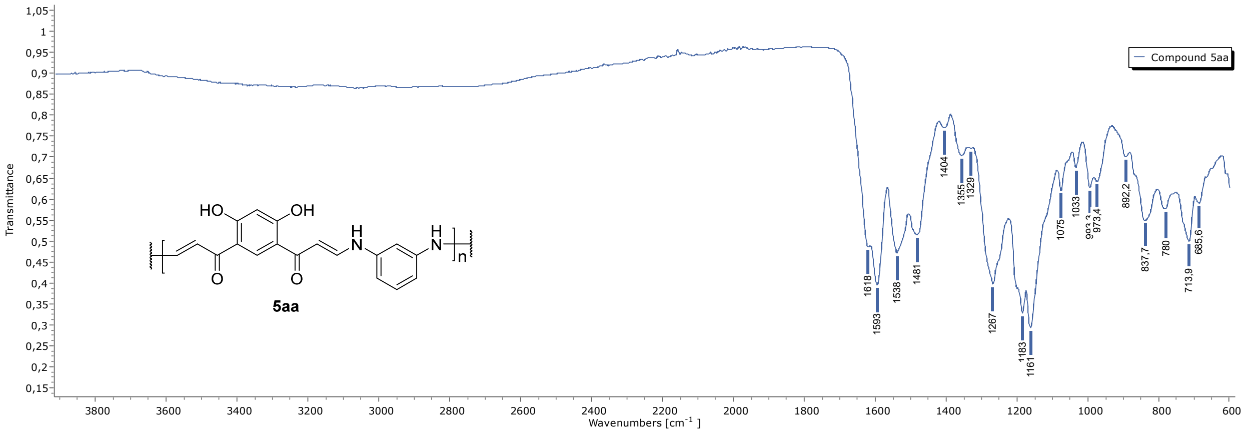
Compound 5aa
Transmittance
Wavenumbers [cm-1]
HO
OH
H
N
N
H
O
O
n
5aa
1618
1593
1538
1481
1404
1355
1329
1267
1183
1161
1075
1033
973,4
892,2
837,7
780
713,9
685,6

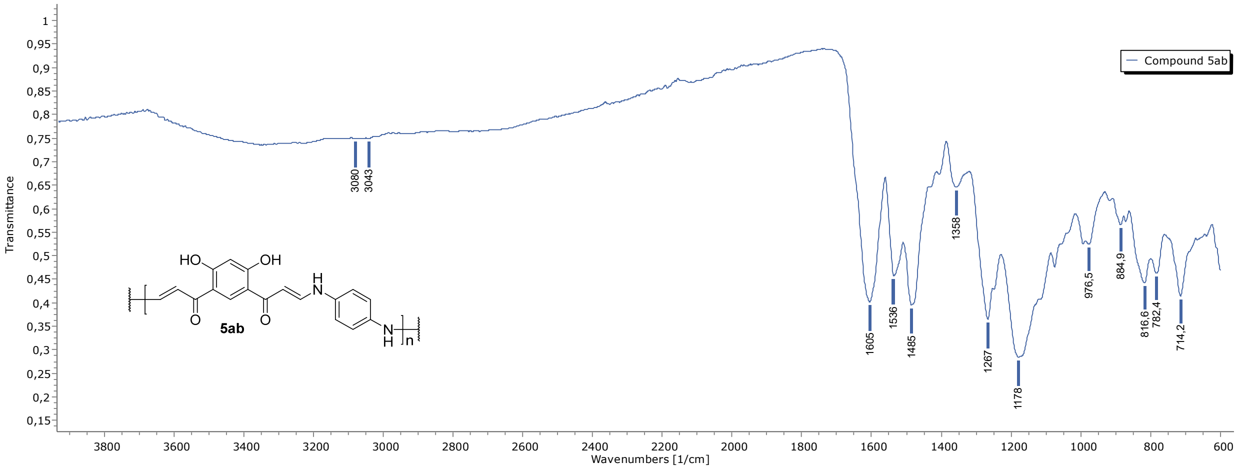
Compound 5ab
Transmittance
Wavenumbers [1/cm]
HO
OH
H
N
N
H
O
O
n
5ab
3080
3043
1605
1536
1485
1358
1267
1178
976,5
884,9
816,6
782,4
714,2

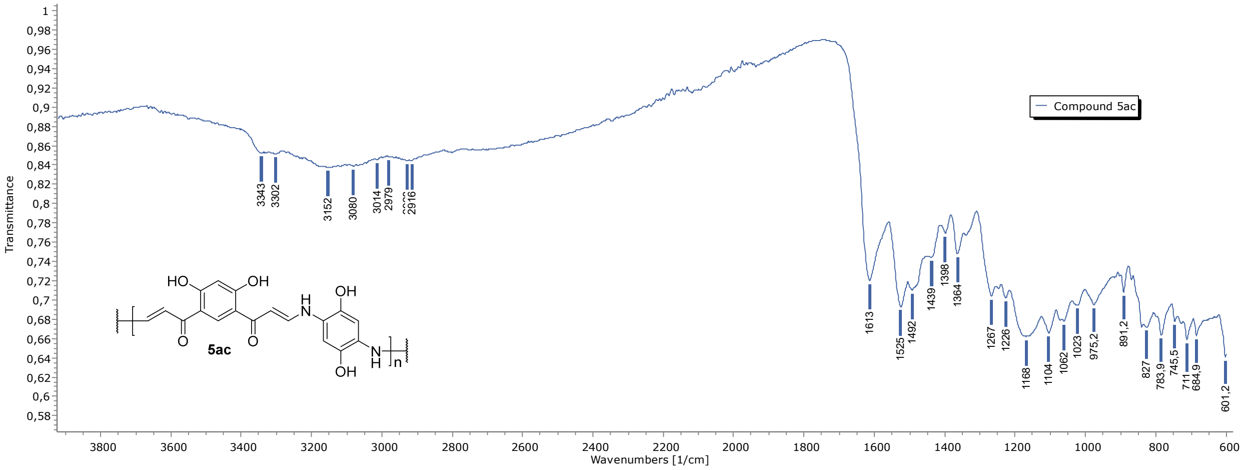
Compound 5ac
Transmittance
Wavenumbers [1/cm]
HO
OH
OH
OH
H
N
N
H
O
O
n
5ac
3343
3302
3152
3080
3014
2979
2916
1613
1525
1492
1439
1398
1364
1267
1226
1168
1104
1062
1023
975,2
891,2
827
783,9
745,5
711
684,9
601,2

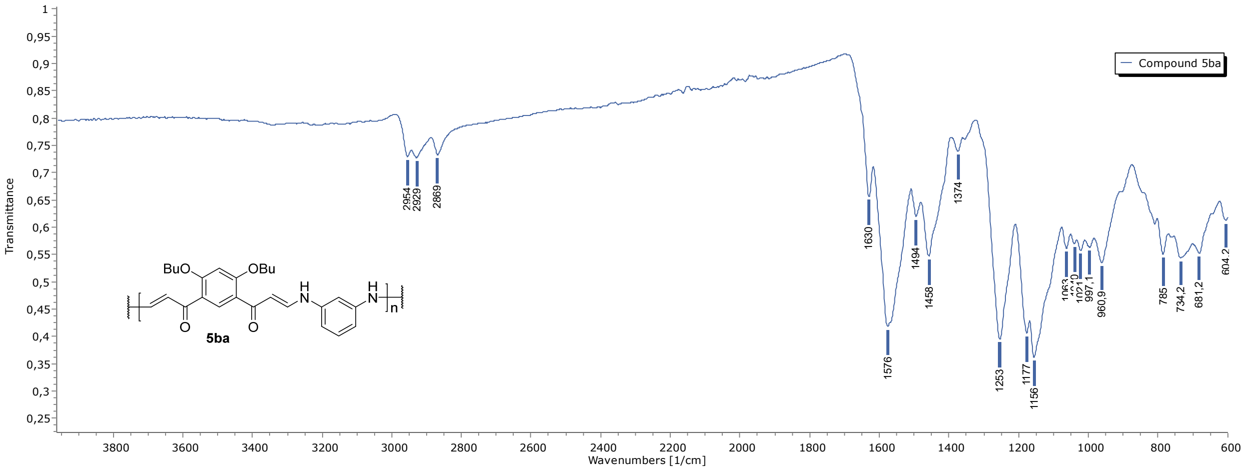
Transmittance
Wavenumbers [1/cm]
Compound 5ba
5ba
BuO
OBu
2954
2929
2869
1630
1576
1494
1458
1374
1253
1177
1156
997,1
960,9
785
734,2
681,2
604,2

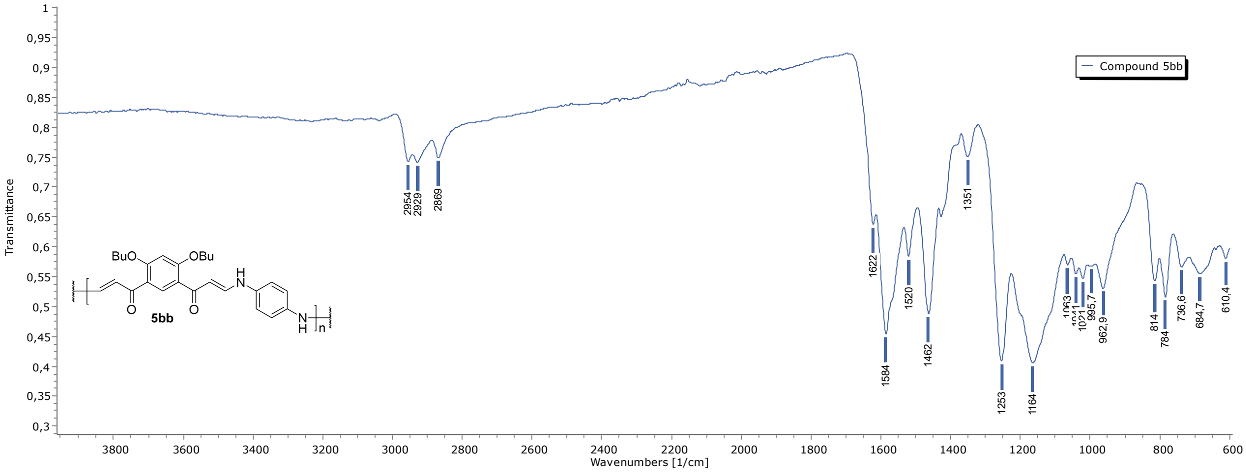
Transmittance
Wavenumbers [1/cm]
Compound 5bb
5bb
BuO
OBu
2954
2929
2869
1622
1584
1520
1462
1351
1253
1164
1063
995,7
962,9
814
784
736,6
684,7
610,4

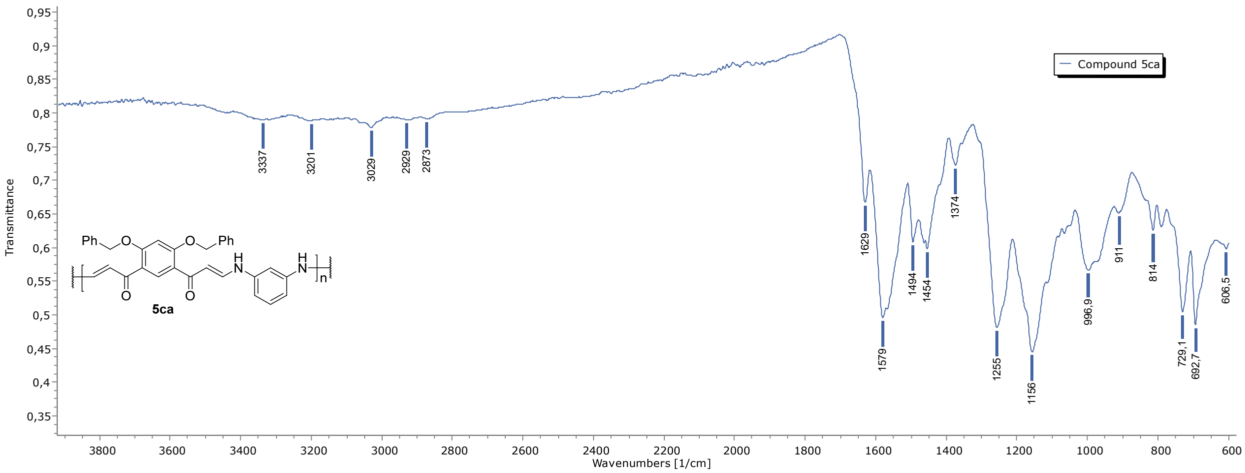
Transmittance
Wavenumbers [1/cm]
Compound 5ca
5ca
Ph
3337
3201
3029
2929
2873
1629
1579
1494
1454
1374
1255
1156
996,9
911
814
729,1
692,7
606,5

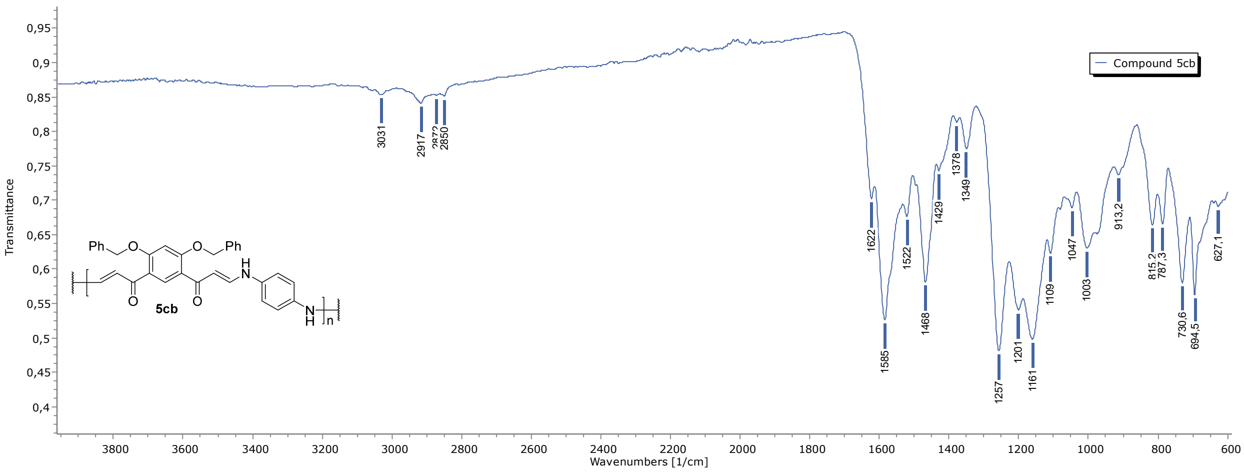
Compound 5cb
Transmittance
Wavenumbers [1/cm]
3031
2917
2872
2850
1622
1585
1522
1468
1429
1378
1349
1257
1201
1161
1109
1047
1003
913,2
815,2
787,3
730,6
694,5
627,1
5cb

3. Copies of UV-VIS spectra of compounds 5.

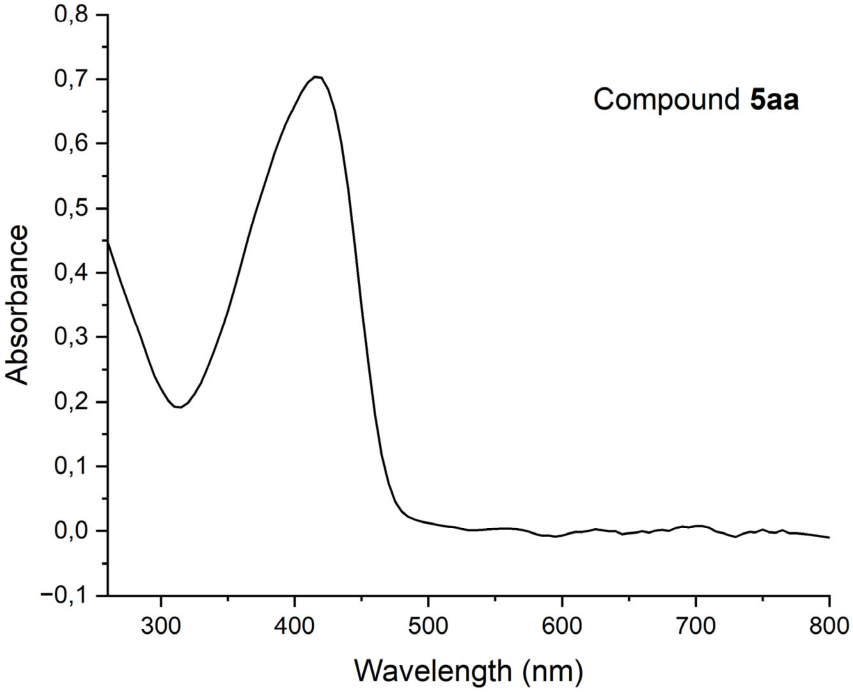

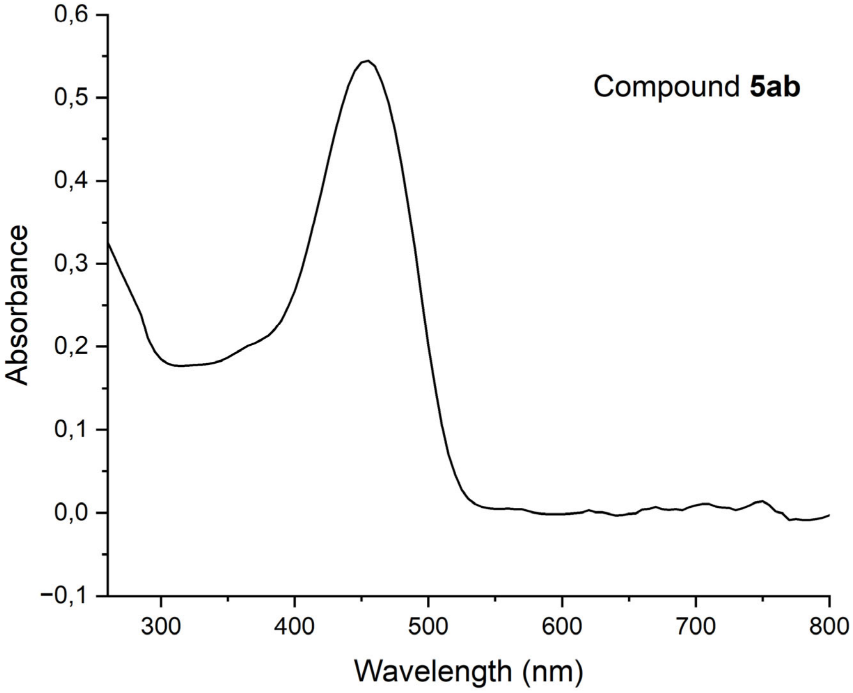

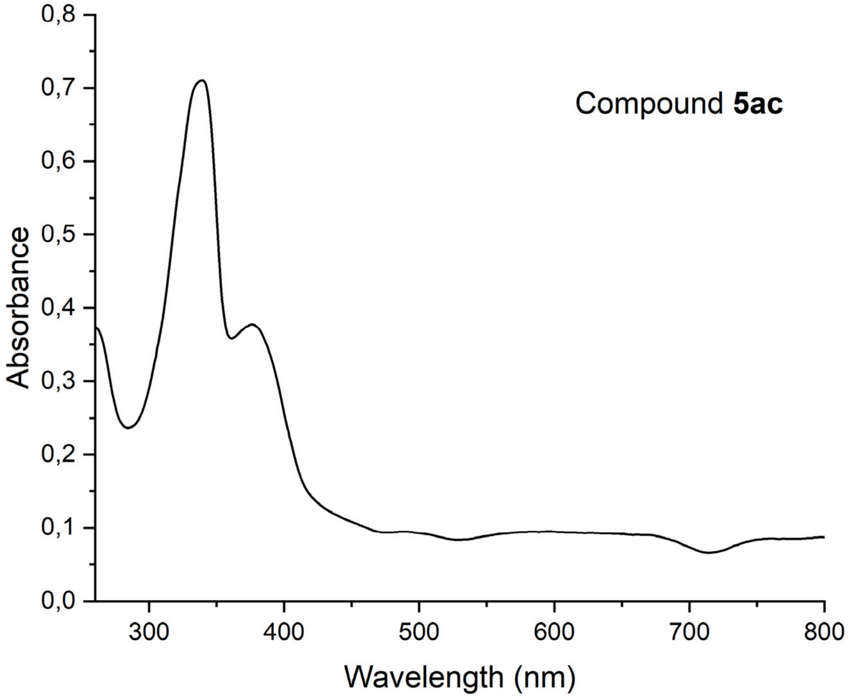
Compound **5ac**
Absorbance
Wavelength (nm)
0,0
0,1
0,2
0,3
0,4
0,5
0,6
0,7
0,8
300
400
500
600
700
800

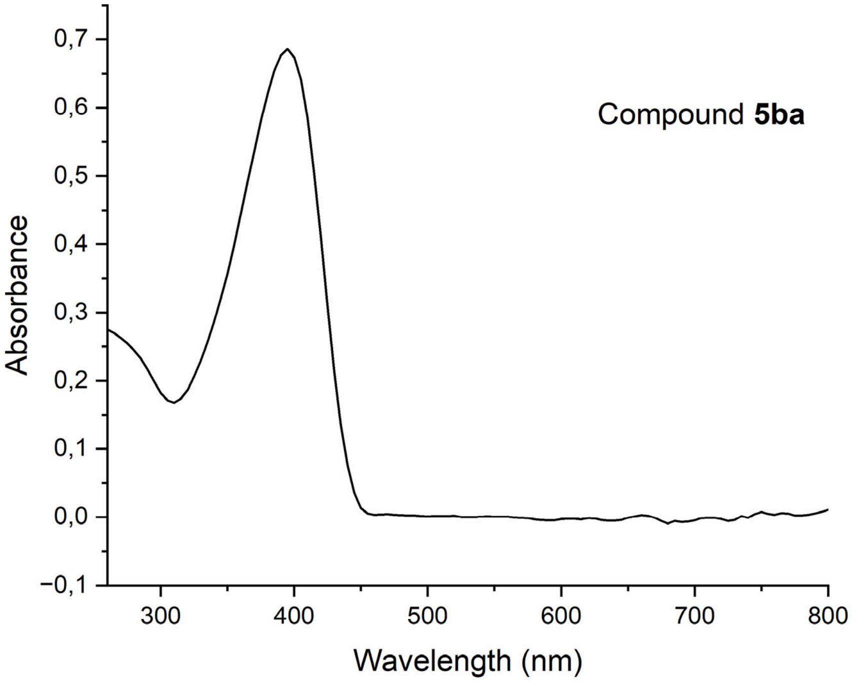
Compound **5ba**
Absorbance
Wavelength (nm)
−0,1
0,0
0,1
0,2
0,3
0,4
0,5
0,6
0,7
300
400
500
600
700
800

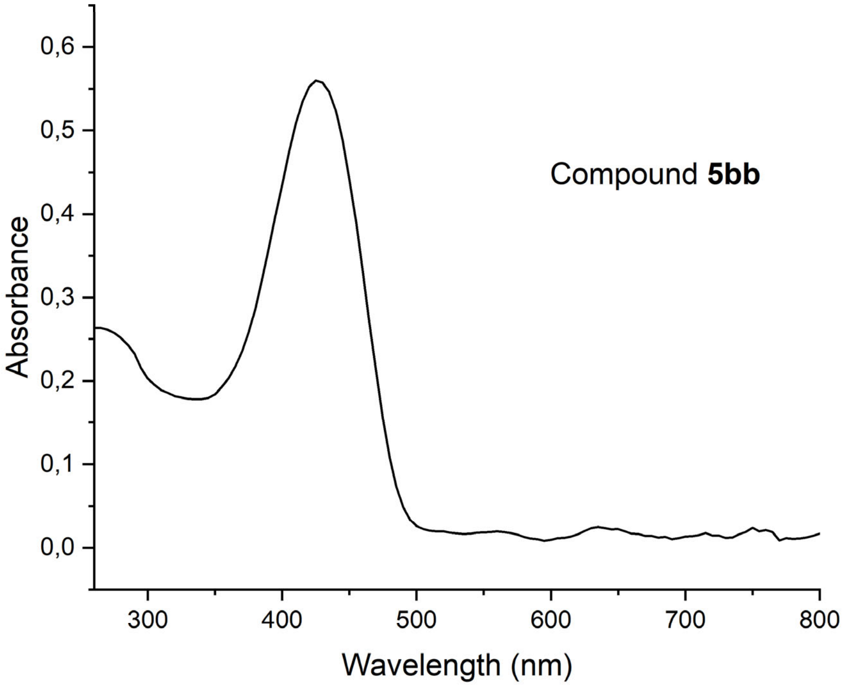
Compound 5bb
Absorbance
Wavelength (nm)
0,6
0,5
0,4
0,3
0,2
0,1
0,0
300
400
500
600
700
800

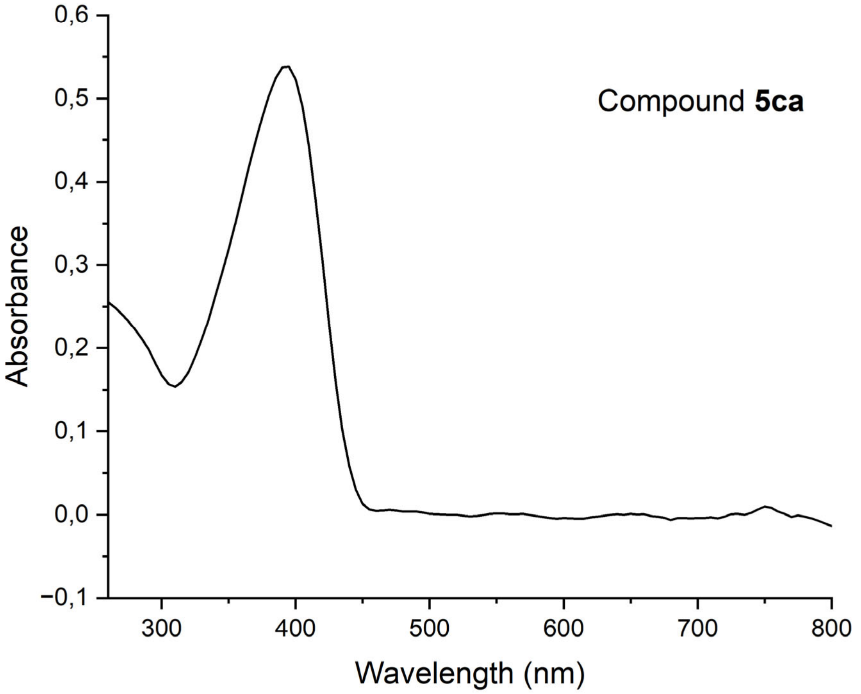
Compound 5ca
Absorbance
Wavelength (nm)
0,6
0,5
0,4
0,3
0,2
0,1
0,0
−0,1
300
400
500
600
700
800

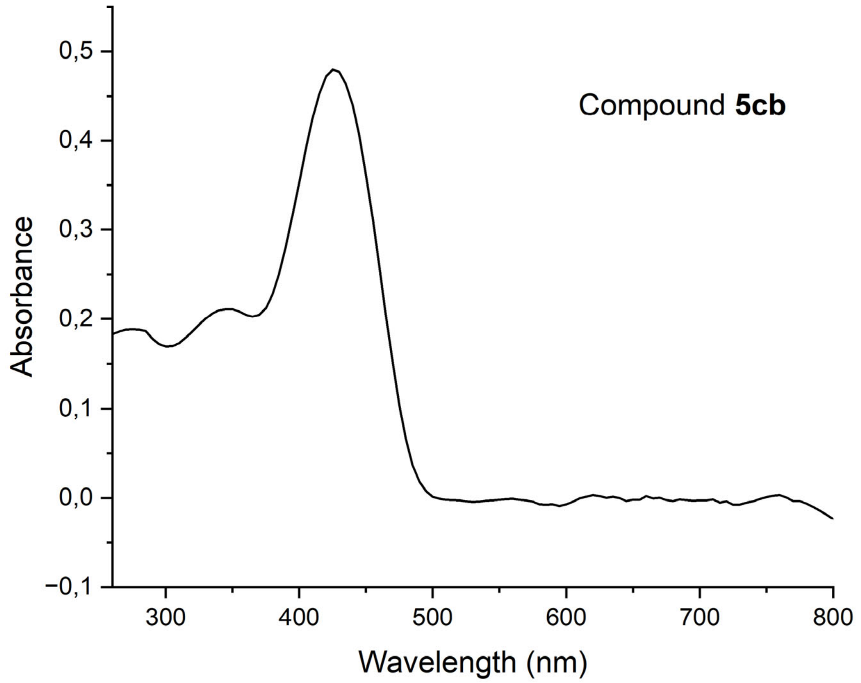

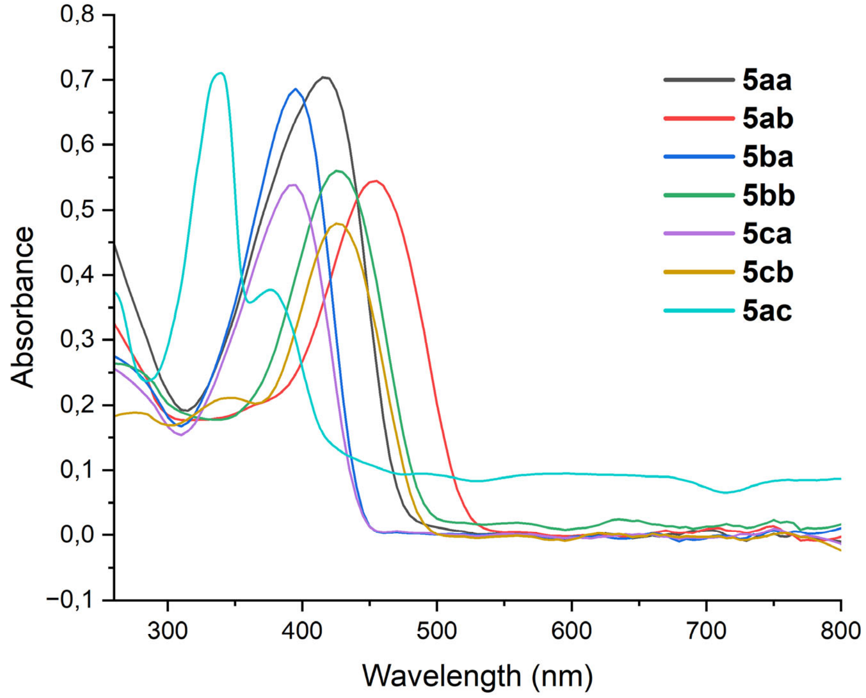

Fig. S1. UV-Vis spectra of compounds **5aa** (—), **5ab** (—), **5ba** (—), **5bb** (—), **5ca** (—), **5cb** (—), and **5ac** (—).

4. Particle size distribution histograms determined from SEM images for polymers 5aa, 5ab, 5ac, 5ba, 5bb, and 5cb

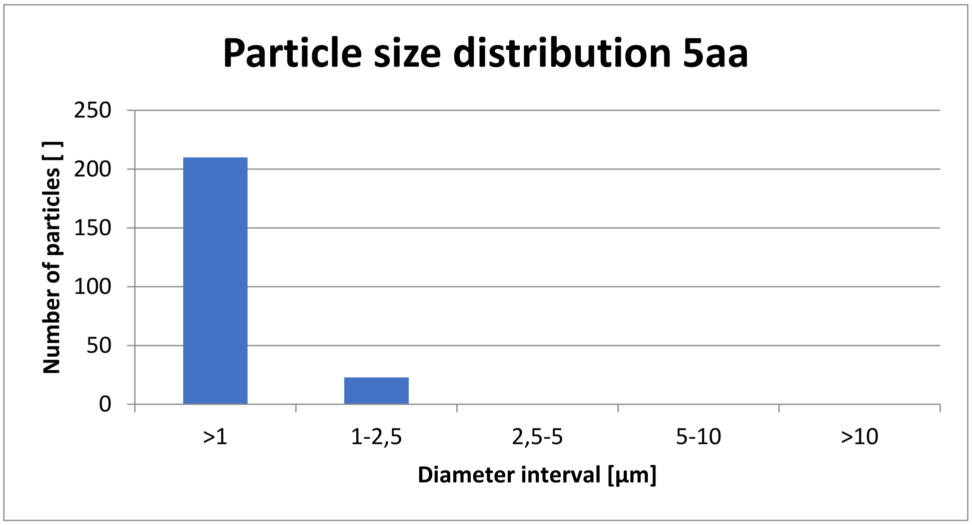

Fig. S2. Particle size distribution histogram determined from SEM image for polymer **5aa**

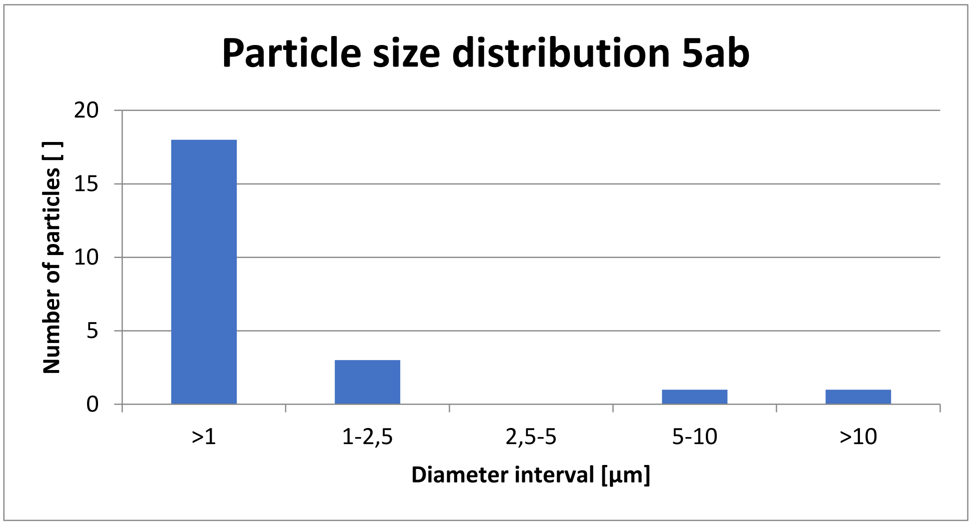

Fig. S3. Particle size distribution histogram determined from SEM image for polymer **5ab**

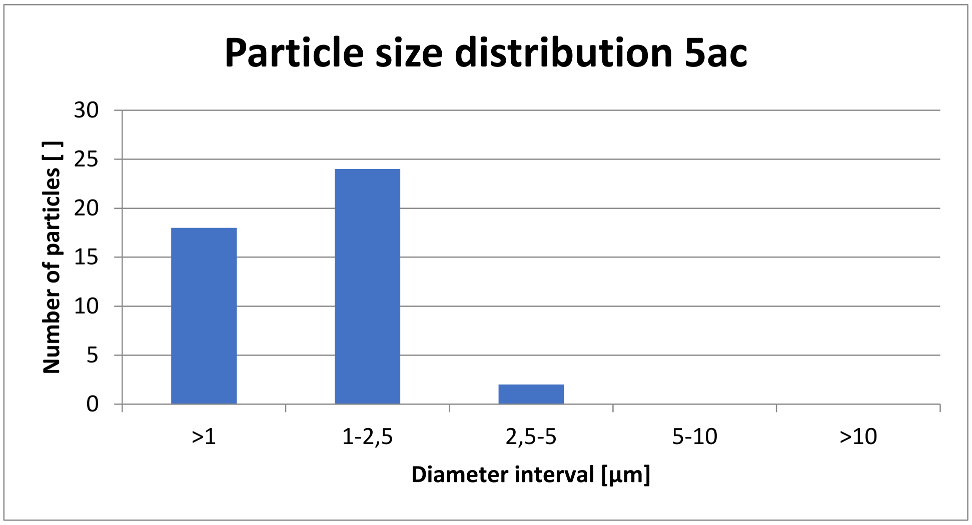

Fig. S4. Particle size distribution histogram determined from SEM image for polymer **5ac**

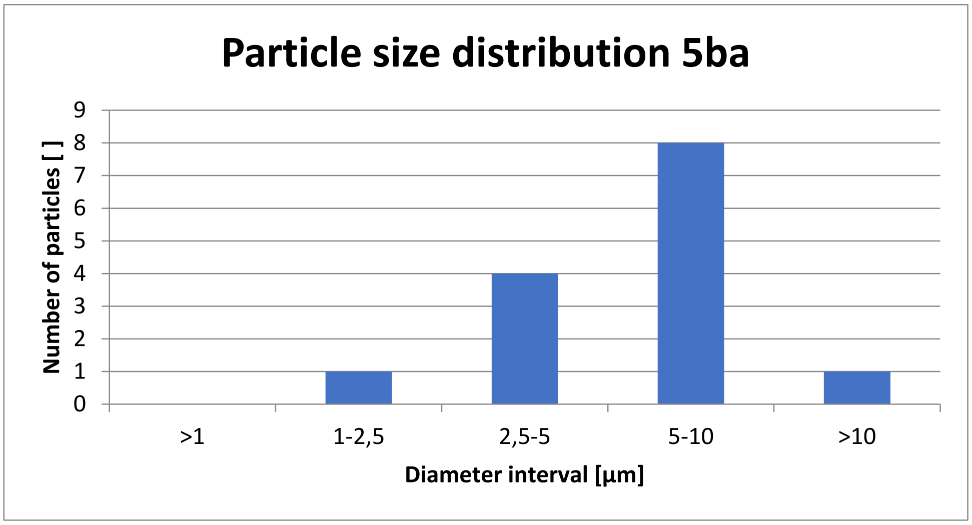

Fig. S5. Particle size distribution histogram determined from SEM image for polymer **5ba**

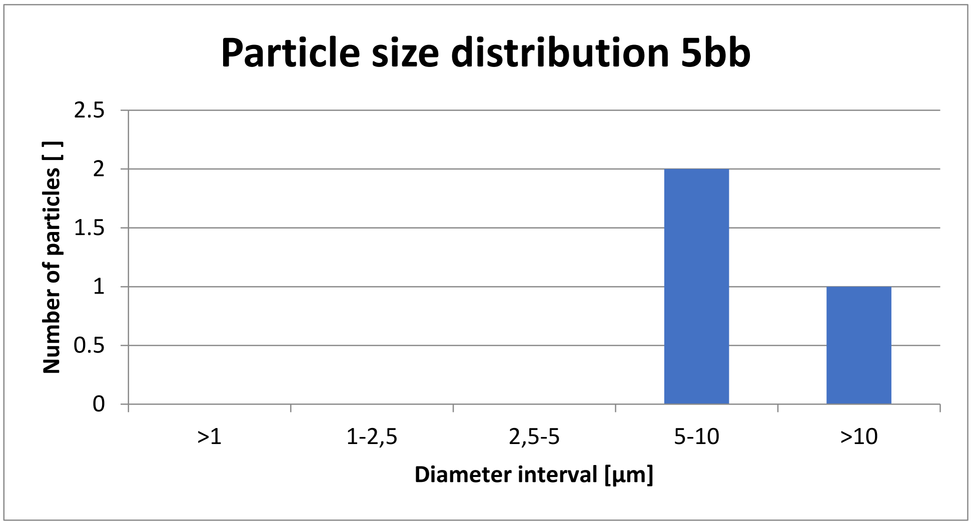

Fig. S6. Particle size distribution histogram determined from SEM image for polymer **5bb**

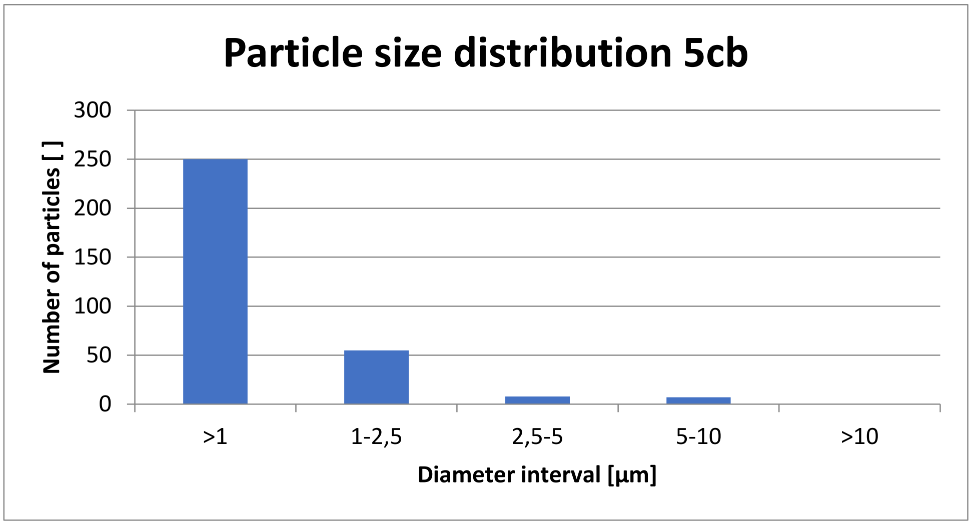

Fig. S7. Particle size distribution histogram determined from SEM image for polymer **5cb**
